# Angel or demon? The dual role of branched-chain amino acids in chronic inflammatory and injury-related diseases

**DOI:** 10.3389/fimmu.2026.1778455

**Published:** 2026-04-22

**Authors:** Jiaxin Li, Hurong Chen, Ying Zhou, Liya Sun, Yao Xing, Yue Sun, Yufeng Yang, Yan Shi

**Affiliations:** Liaoning University of Traditional Chinese Medicine, Shenyang, China

**Keywords:** branched-chain amino acids (BCAAs), insulin resistance, liver cirrhosis, metabolic homeostasis, mTORC1 signaling, precision nutrition

## Abstract

Branched-chain amino acids (BCAAs)—leucine, isoleucine, and valine—are essential nutrients that exhibit context-dependent, paradoxical effects on human health, with mTORC1 (mechanistic target of rapamycin complex 1) signaling serving as a central mechanistic node through which physiological BCAA concentrations support anabolism and repair while chronic pathological elevation drives metabolic and inflammatory injury. While their anabolic properties in promoting muscle protein synthesis, modulating immune responses, and conferring hepatoprotection are well-documented, accumulating evidence demonstrates that chronically elevated circulating BCAA concentrations are strongly associated with the pathogenesis and progression of metabolic, inflammatory, and injury-related diseases, including insulin resistance, type 2 diabetes mellitus (T2DM), cardiovascular disease (CVD), metabolic dysfunction-associated steatotic liver disease (MASLD, formerly NAFLD), and certain malignancies. This biological duality is mechanistically rooted in a network of interconnected pathological processes, in which BCAA-mediated modulation of mTORC1 signaling—already introduced above—represents one central hub operating alongside impaired catabolic flux, accumulation of branched-chain α-keto acids (BCKAs) and branched-chain acylcarnitines, mitochondrial redox imbalance, and cellular stress pathway activation. Physiological BCAA concentrations support anabolic processes and cellular repair, whereas chronic pathological elevation is associated with mTORC1 hyperactivation alongside impaired BCKDH-mediated catabolic flux, accumulation of branched-chain α-keto acids (BCKAs) and branched-chain acylcarnitines, mitochondrial redox imbalance, and activation of cellular stress pathways—collectively contributing to disrupted metabolic homeostasis, amplified inflammatory cascades, and mitochondrial dysfunction. The ultimate biological impact of BCAAs is not intrinsic to these amino acids but rather is determined by a complex interplay of factors including: dosage and duration of exposure, individual metabolic status (particularly insulin sensitivity and mitochondrial oxidative capacity), specific disease context, and genetic polymorphisms affecting BCAA metabolism alongside gut microbiome composition. This review comprehensively synthesizes current understanding of BCAA biology and advocates for a paradigm shift toward precision nutrition approaches. Evidence supports therapeutic BCAA supplementation in hypercatabolic conditions such as sarcopenia and hepatic cirrhosis, while suggesting potential adverse metabolic consequences in insulin-resistant or obese individuals. Future nutritional and therapeutic strategies should transition from universal dietary recommendations to personalized interventions based on comprehensive metabolic phenotyping and genetic profiling, thereby optimizing BCAA intake for individual health trajectories and providing novel preventive and therapeutic opportunities for chronic disease management.

## 1 Introduction

Branched-chain amino acids (BCAAs)—leucine, isoleucine, and valine—are essential amino acids characterized by aliphatic side chains with branched structures (1). These amino acids constitute approximately 35-40% of total essential amino acid intake and represent 18-20% of skeletal muscle protein composition (1). Unlike other amino acids undergoing hepatic first-pass metabolism, BCAAs are primarily metabolized in peripheral tissues, particularly skeletal muscle and adipose tissue (2).This unique metabolic characteristic endows BCAAs with specialized roles in energy metabolism and protein synthesis.

The physiological functions of BCAAs extend beyond serving as protein building blocks. They critically activate the mammalian target of rapamycin complex 1 (mTORC1), orchestrating cellular growth, proliferation, and metabolism (3, 4). Additionally, BCAAs function as energy substrates during prolonged exercise or starvation, supplying intermediates for the tricarboxylic acid cycle (5). Other biological activities include participation in neurotransmitter synthesis, immune function regulation, and glucose homeostasis maintenance (2, 5).

Chronic inflammatory and injury-related diseases represent conditions characterized by persistent, low-grade inflammation and dysregulated tissue repair. This spectrum encompasses metabolic disorders (type 2 diabetes, metabolic dysfunction-associated steatotic liver disease), autoimmune diseases (rheumatoid arthritis, inflammatory bowel disease), cardiovascular diseases, and neurodegenerative disorders (6). According to the Global Burden of Disease study, chronic non-communicable diseases account for 74% of global mortality, with chronic inflammation recognized as their common pathological foundation (7). The global diabetic population has surpassed 537 million and is projected to reach 783 million by 2045 (8).

In this review, “metabolic, inflammatory, and injury-related diseases” is used as a broad categorical term. It encompasses conditions driven primarily by metabolic dysregulation (such as insulin resistance and type 2 diabetes), those characterized by persistent inflammatory activation (such as rheumatoid arthritis and inflammatory bowel disease), and those in which chronic tissue injury is the central pathological feature (such as liver cirrhosis and hepatic encephalopathy). Liver cirrhosis and hepatic encephalopathy are included in this framework because chronic hepatic parenchymal injury and neuroinflammation—rather than canonical systemic inflammation—constitute their core pathophysiological mechanisms. This classification reflects the mechanistic heterogeneity of conditions in which BCAA metabolism is implicated, rather than implying a uniform inflammatory etiology.

Clinical application of BCAAs began in the 1970s, initially focusing on nutritional support for critically ill patients. Subsequently, BCAA supplementation was found to significantly improve hepatic encephalopathy in cirrhotic patients by competitively inhibiting aromatic amino acid brain entry (9). However, in 2009, Newgard and colleagues published a landmark study demonstrating that plasma BCAA levels were significantly elevated in obese and insulin-resistant individuals, and that elevated fasting circulating BCAA concentrations predicted future type 2 diabetes development (10). This discovery challenged the traditional perception of BCAAs as uniformly beneficial nutrients.

Subsequent investigations revealed associations between elevated BCAAs and cardiovascular disease, MASLD, and cancer (11, 12). Over the past decade, researchers have increasingly recognized the dual nature of BCAAs. On one hand, BCAA supplementation improves liver function and survival in cirrhotic patients, helps maintain muscle mass in cachexia, and exhibits immunomodulatory effects (9, 13). On the other hand, prospective cohort studies indicate positive correlations between plasma BCAAs and risks of type 2 diabetes, cardiovascular disease, and cancer; high-BCAA diets induce insulin resistance in experimental animals; and BCAA restriction extends lifespan in laboratory animals (14–16). These contradictory findings have sparked intense academic debate regarding whether elevated BCAAs are causes or consequences of disease, and how to explain heterogeneous effects across different populations (14, 17). Mechanistically, the pathological effects of BCAAs are not attributable to a single molecular switch but rather emerge from a broader network that includes mTORC1 as one major hub, alongside impaired catabolic flux, toxic accumulation of BCKAs and branched-chain acylcarnitines, mitochondrial redox imbalance, and cellular stress pathway activation; where underlying evidence is associative rather than interventional, causal interpretations are presented with appropriate qualification throughout this review.

This review systematically examines the dual role of BCAAs in chronic inflammatory and injury-related diseases through a context-dependent mechanistic framework centered on mTORC1 signaling. We summarize BCAA metabolic pathways, elucidate both protective and pathogenic mechanisms with particular attention to the distinction between physiological and pathological mTORC1 activation states, and analyze disease-specific clinical implications and intervention strategies. Through this analysis, we aim to provide comprehensive perspectives for nutritional management of chronic inflammatory diseases and establish theoretical foundations for novel therapeutic strategies targeting BCAA metabolism.

## 2 Metabolism and regulation of BCAAs

### 2.1 Metabolic pathways of branched-chain amino acids

**Figure 1:**
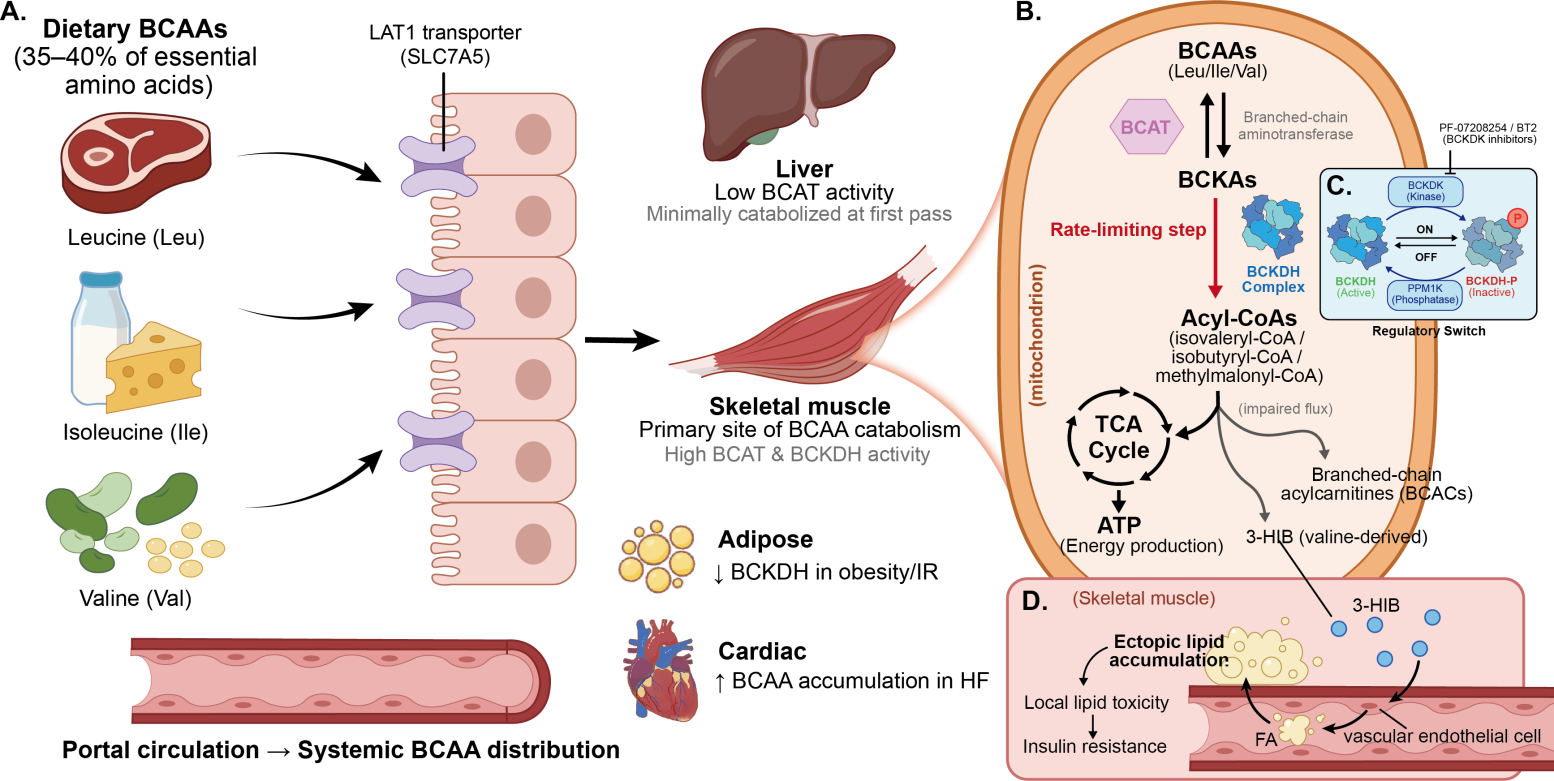
BCAA metabolic pathways: from dietary intake to tissue-specific catabolism and the BCKDH regulatory switch This figure illustrates BCAA metabolism from systemic distribution to mitochondrial catabolism, with emphasis on BCKDH regulatory mechanisms and the corrected 3-HIB mechanism. **(A)** Tissue-level distribution. Dietary BCAAs are absorbed via the LAT1 transporter (SLC7A5) and distributed systemically. The liver contributes minimally to initial BCAA catabolism due to low BCAT expression. Skeletal muscle serves as the primary catabolic site. Adipose tissue shows reduced BCKDH activity in obesity and insulin resistance, and cardiac tissue exhibits BCAA accumulation in heart failure. **(B)** Mitochondrial catabolic pathway. BCAA catabolism proceeds through two enzymatic steps: reversible transamination by BCAT generating BCKAs, followed by irreversible oxidative decarboxylation by the BCKDH complex (rate-limiting step) yielding acyl-CoA derivatives that enter the TCA cycle. When catabolic flux is impaired, branched-chain acylcarnitines (BCACs) accumulate and exert independent pathological effects. **(C)** The regulatory switch. BCKDH activity is controlled by a phosphorylation/dephosphorylation cycle: BCKDK phosphorylates and inactivates BCKDH (“OFF”), while PPM1K dephosphorylates and activates it (“ON”). Pharmacological BCKDK inhibitors (PF-07208254, BT2) maintain BCKDH in its active state to promote BCAA disposal, though long-term human safety data remain to be established. **(D)** Corrected 3-HIB mechanism. The valine-derived metabolite 3-HIB is secreted from skeletal muscle and enhances trans-endothelial fatty acid transport, promoting ectopic lipid accumulation within skeletal muscle—not transport to the liver—thereby contributing to local lipotoxicity and insulin resistance. BCAA, Branched-Chain Amino Acid; LAT1, L-Type Amino Acid Transporter 1; SLC7A5, Solute Carrier Family 7 Member 5; Leu, Leucine; Ile: Isoleucine; Val, Valine; BCAT, Branched-Chain Amino Acid Transaminase; BCKDH, Branched-Chain Alpha-Keto Acid Dehydrogenase; TCA, Tricarboxylic Acid Cycle; BCACs, Branched-Chain Acylcarnitines; 3-HIB, 3-Hydroxyisobutyrate; BCKDK, Branched-Chain Alpha-Keto Acid Dehydrogenase Kinase; PPM1K, Protein Phosphatase Mg2+/Mn2+ Dependent 1K; HF, Heart Failure; IR, Insulin Resistance; ATP, Adenosine Triphosphate.

As essential amino acids, branched-chain amino acids (BCAAs) must be obtained through dietary intake, primarily from protein-rich foods such as meat, dairy products, and legumes. Their intestinal absorption is predominantly mediated by L-type amino acid transporter 1 (LAT1), followed by systemic distribution via blood circulation to various tissues throughout the body (18). Unlike other amino acids that are primarily metabolized in the liver, BCAAs exhibit a unique metabolic characteristic: their initial catabolic reactions predominantly occur in extrahepatic tissues, particularly skeletal muscle (5). (The tissue-level distribution of BCAA metabolism across skeletal muscle, adipose tissue, liver, and cardiac tissue is illustrated in **Figure 1A**. The catabolism of BCAAs follows a highly conserved two-step enzymatic cascade. The mitochondrial catabolic pathway—from BCAT-mediated transamination to BCKDH-mediated oxidative decarboxylation—is depicted in **Figure 1B**, and the BCKDK/PPM1K regulatory phosphorylation switch governing BCKDH activity is shown in **Figure 1C**. see **Figure 1D** for corrected 3-HIB mechanism).

The catabolism of BCAAs follows a highly conserved two-step enzymatic cascade. The first step involves a reversible transamination reaction catalyzed by branched-chain aminotransferase (BCAT), generating the corresponding branched-chain α-keto acids (BCKAs). The second step comprises an irreversible oxidative decarboxylation reaction catalyzed by the branched-chain α-keto acid dehydrogenase complex (BCKDH), which represents the rate-limiting step of the entire metabolic pathway and directs metabolism toward complete oxidation (19). BCKDH activity is subject to sophisticated post-translational modification: branched-chain α-keto acid dehydrogenase kinase (BCKDK) inactivates it through phosphorylation, while mitochondrial phosphatase PPM1K activates it through dephosphorylation (20). This regulatory mechanism enables BCKDH to rapidly respond to changes in nutritional status and energy demands. In recent years, small molecule inhibitors targeting BCKDK have emerged as a research hotspot. For instance, the thiophene compound PF-07208254 not only inhibits BCKDK enzymatic activity but also promotes its protein degradation, thereby sustainably reducing BCKAs levels and improving cardiovascular metabolic parameters in mice (21). This finding provides novel insights for developing therapies targeting BCAA metabolism.

It is important to emphasise, however, that the existing evidence for BCKDK inhibition as a therapeutic strategy derives predominantly from preclinical animal models, and direct translation to human clinical practice requires caution. Since BCAAs are essential amino acids that cannot be synthesised endogenously, sustained pharmacological enhancement of BCAA catabolism carries theoretical risks of reducing BCAA availability below levels required for muscle protein synthesis, neurological function, and other critical physiological processes. Populations with high anabolic demand—including athletes, growing children, malnourished patients, and those with sarcopenia—may be particularly vulnerable to iatrogenic BCAA insufficiency. Furthermore, the long-term safety profile of BCKDK inhibitors in humans, optimal dosing windows, tissue-specific off-target effects, and patient-specific contraindications remain to be established in adequately powered and duration-sufficient clinical trials. Current pharmacological data should therefore be interpreted as hypothesis-generating rather than practice-changing.

**Figure 2:**
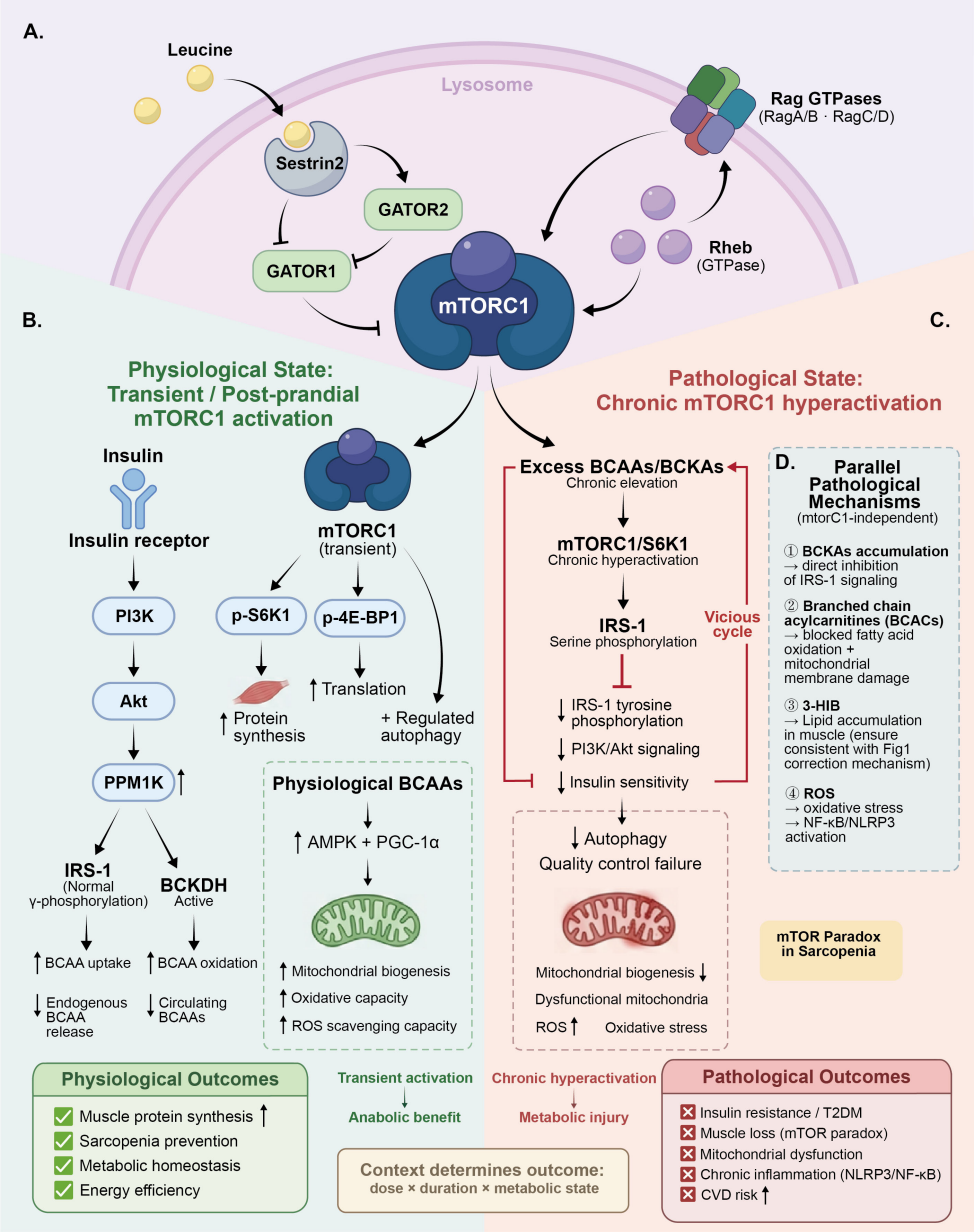
mTORC1 as a central signaling node in BCAA biology: physiological anabolism versus pathological metabolic dysregulation. This figure presents an integrated view of the mechanistic distinction between physiological transient mTORC1 activation and chronic pathological hyperactivation, alongside mTORC1-independent parallel pathological mechanisms. **(A)** Leucine sensing and mTORC1 activation. Leucine binding to Sestrin2 relieves inhibition of GATOR2, activating the Rag GTPase complex and recruiting mTORC1 to the lysosomal surface for Rheb-mediated activation. Activated mTORC1 phosphorylates S6K1 and 4E-BP1, promoting protein synthesis and regulating autophagy flux. **(B)** Physiological state: transient mTORC1 activation. Under insulin-sensitive conditions, insulin activates PPM1K via PI3K–Akt signaling, enhancing BCKDH activity and promoting BCAA uptake and oxidation to maintain circulating BCAA homeostasis. Physiological BCAA concentrations additionally stimulate mitochondrial biogenesis through AMPK–PGC-1α, supporting oxidative capacity and ROS scavenging. Net outcomes include enhanced muscle protein synthesis, sarcopenia prevention, and metabolic homeostasis. **(C)** Pathological state: chronic mTORC1 hyperactivation. Chronically elevated BCAAs/BCKAs—reflecting impaired catabolic disposal rather than excess dietary intake—drive sustained mTORC1/S6K1 activation, leading to IRS-1 serine phosphorylation, impaired PI3K/Akt signaling, and reduced insulin sensitivity. This establishes a self-perpetuating vicious cycle of BCAA accumulation and insulin resistance, with downstream consequences including autophagy failure, mitochondrial dysfunction, and oxidative stress. Pathological outcomes encompass insulin resistance/T2DM, muscle loss (mTOR paradox), chronic inflammation (NLRP3/NF-κB), and elevated CVD risk. **(D)** Parallel mTORC1-independent mechanisms. mTORC1 hyperactivation represents one major hub within a broader pathological network. Additional mechanisms include: 1. BCKA accumulation → direct IRS-1 signal inhibition;2. BCACs → impaired fatty acid oxidation and mitochondrial membrane damage; 3. 3-HIB → ectopic lipid accumulation in skeletal muscle (see **Figure 1D**); 4. ROS → NF-κB/NLRP3 activation. The mTOR paradox in sarcopenia is highlighted: impaired mitochondrial BCAA catabolism in aged muscle paradoxically drives mTORC1 hyperactivation, causing muscle atrophy rather than growth. mTORC1, Mechanistic Target of Rapamycin Complex 1; Rag, Rag GTPase; GATOR1, GTPase-Activating Protein Toward Rags 1; GATOR2, GTPase-Activating Protein Toward Rags 2; Rheb, Ras Homolog Enriched in Brain; S6K1, Ribosomal Protein S6 Kinase Beta-1; 4E-BP1, Eukaryotic Translation Initiation Factor 4E-Binding Protein 1; IRS-1, Insulin Receptor Substrate 1; PI3K, Phosphoinositide 3-Kinase; Akt, Protein Kinase B; PPM1K, Protein Phosphatase, Mg2+/Mn2+ Dependent 1K; AMPK, AMP-Activated Protein Kinase; PGC-1α, Peroxisome Proliferator-Activated Receptor Gamma Coactivator 1-Alpha; ROS, Reactive Oxygen Species; NF-κB, Nuclear Factor Kappa-Light-Chain-Enhancer of Activated B Cells; NLRP3, NLR Family Pyrin Domain Containing 3; BCKA, Branched-Chain Keto Acid; BCACs, Branched-Chain Acylcarnitines; 3-HIB, 3-Hydroxyisobutyrate; T2DM, Type 2 Diabetes Mellitus; CVD, Cardiovascular Disease.

As shown in **Figure 2**, BCAA metabolites possess diverse biological functions under both physiological and pathological conditions. As energy substrates, BCAAs can serve as alternative energy sources during glycogen depletion, with their oxidative products replenishing tricarboxylic acid cycle intermediates and maintaining energy homeostasis (22). However, when metabolic pathways are impaired, the accumulation of intermediate products may exert toxic effects. Notably, 3-hydroxyisobutyrate (3-HIB), is secreted from skeletal muscle and acts on the vascular endothelium to enhance trans-endothelial fatty acid transport, promoting fatty acid uptake and ectopic lipid accumulation within skeletal muscle itself, thereby contributing to local lipotoxicity and insulin resistance (23). In hereditary maple syrup urine disease, severe accumulation of BCAAs and BCKAs directly causes neurotoxicity and metabolic acidosis (24).

Precision in terminology is essential for interpreting BCAA research and avoiding conflation of mechanistically distinct entities. Throughout this review, four conceptually separate constructs are consistently distinguished. First, dietary BCAA intake refers to the quantity of BCAAs consumed from food sources as assessed by dietary tools, reflecting habitual protein consumption patterns rather than metabolic status. Second, BCAA supplementation refers to exogenous administration at defined doses, in specific formulations (free amino acids, peptide-bound forms, or leucine-enriched blends), via designated routes (oral, enteral, or parenteral). Third, circulating BCAA concentrations—typically measured as fasting plasma levels—reflect the net balance of dietary input, endogenous release from proteolysis, tissue uptake, and catabolic clearance. Critically, elevated fasting circulating BCAAs in obesity and insulin resistance primarily indicate impaired peripheral catabolic disposal and altered inter-organ flux, not simply excessive dietary intake; this distinction carries fundamental implications for intervention strategy. Fourth, catabolic intermediates—branched-chain α-keto acids (BCKAs) and branched-chain acylcarnitines (BCACs)—accumulate when BCKDH flux is impaired and exert independent pathological effects that are mechanistically distinct from those of BCAAs per se. Disease-specific sections of this review apply this framework consistently.

### 2.2 Key regulatory nodes of metabolism

#### 2.2.1 Activation of the mTORC1 signaling pathway

Leucine is recognized as the most potent natural mTORC1 activator, with its sensing and signal transduction mechanisms involving a complex sensor network. Key steps include leucine binding to the Sestrin2 protein, relieving its inhibition of the GATOR2 complex, subsequently promoting Rag GTPase complex activation, and ultimately recruiting mTORC1 to the lysosomal surface for activation by the Rheb protein (25, 26). Activated mTORC1 promotes protein synthesis and suppresses autophagy through phosphorylation of downstream targets S6K1 and 4E-BP1, thereby coordinating the balance between anabolic and catabolic metabolism (27). It should be noted that mTORC1 represents one major signaling hub within a broader pathological network rather than a singular unifying switch; its activation is therefore described throughout this review as contributing to or being associated with downstream metabolic consequences, rather than as a sole causal determinant. Notably, different amino acid combinations exhibit varying effects on mTORC1 activation. Research demonstrates that compared to leucine alone, BCAA mixtures more potently activate S6K1 following exercise, while mixtures containing all essential amino acids yield optimal effects, suggesting that a complete amino acid profile maximizes mTORC1 activation and protein synthesis responses (28).

**Figure 3:**
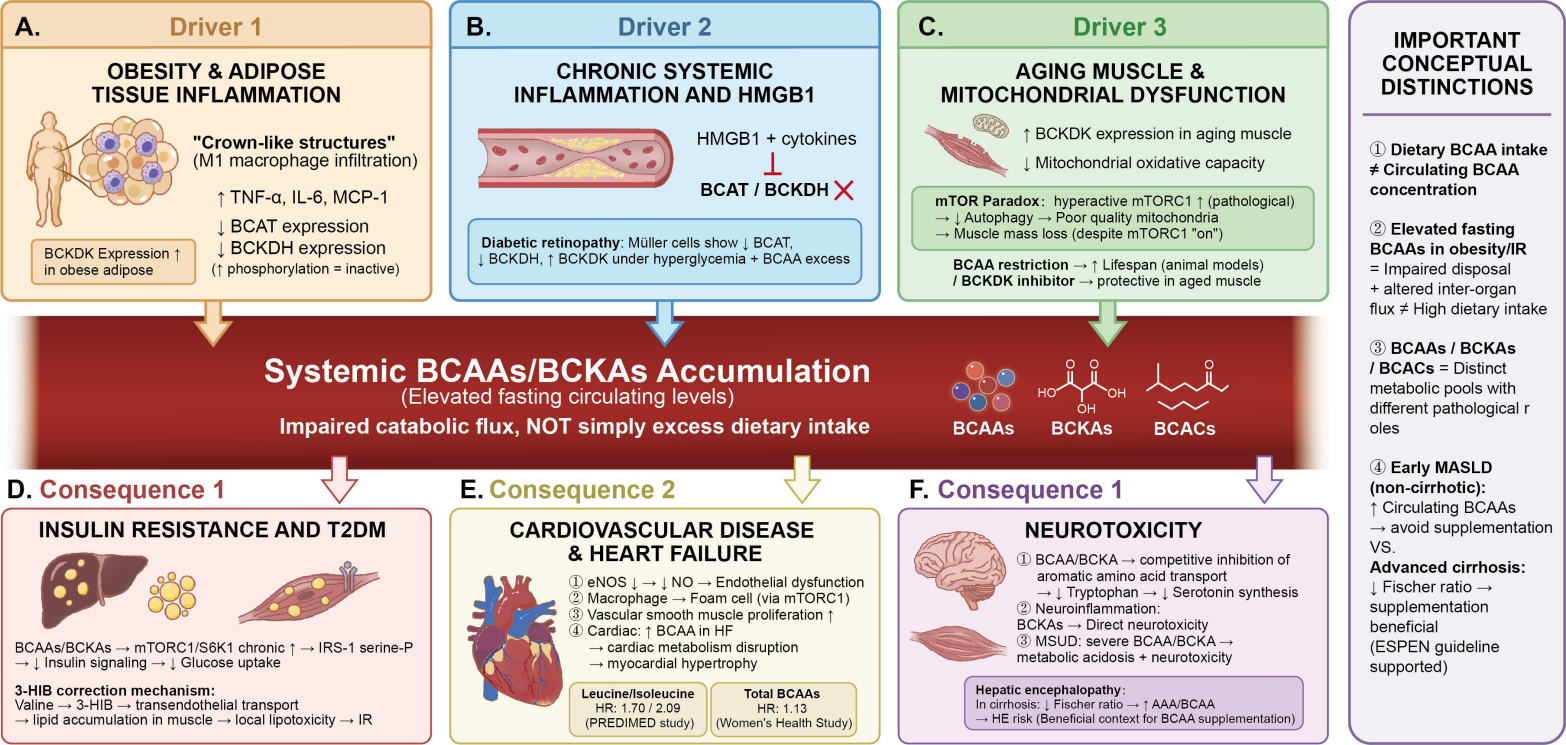
Upstream drivers of systemic BCAA/BCKA accumulation and downstream multi-organ pathological consequences. This figure employs a convergence–divergence architecture to delineate three major upstream pathological drivers of systemic BCAA/BCKA accumulation and the resulting multi-organ downstream consequences, with a conceptual distinctions panel to guide clinical interpretation. **(A)** Driver 1: obesity and adipose tissue inflammation. Adipose expansion triggers M1 macrophage infiltration (“crown-like structures”) and pro-inflammatory cytokine release (TNF-α, IL-6, MCP-1), transcriptionally repressing BCAT and BCKDH expression while upregulating BCKDK, thereby impairing systemic BCAA clearance. The resulting fasting BCAA elevation reflects impaired disposal and altered inter-organ flux—not simply excess dietary intake. **(B)** Driver 2: chronic systemic inflammation and HMGB1. HMGB1 released under tumor burden or cellular stress further suppresses BCAT and BCKDH expression. In diabetic retinopathy, hyperglycemia combined with BCAA excess reduces BCAT and BCKDH in Müller cells while elevating inflammatory mediators. **(C)** Driver 3: aging muscle and mitochondrial dysfunction. Age-related decline in mitochondrial oxidative capacity generates the “mTOR paradox”: impaired BCAA catabolism → BCAA accumulation → pathological mTORC1 hyperactivation → autophagy suppression and mitochondrial deterioration → muscle atrophy rather than growth. Elevated BCKDK expression in aging compounds this derangement. BCAA restriction or BCKDK inhibition is protective in aged muscle models. (Central nexus.) Systemic BCAA/BCKA accumulation (elevated fasting circulating levels) arises from impaired catabolic flux—not simply excess dietary intake. **(D)** Consequence 1: insulin resistance and T2DM. BCAAs/BCKAs → chronic mTORC1/S6K1 activation → IRS-1 serine phosphorylation → impaired insulin signaling → reduced glucose uptake. 3-HIB (secreted from muscle) enhances trans-endothelial fatty acid transport → ectopic lipid accumulation in skeletal muscle → local lipotoxicity → insulin resistance. **(E)** Consequence 2: cardiovascular disease and heart failure. Endothelial dysfunction (eNOS↓ → NO↓), vascular smooth muscle cell proliferation, mTORC1-driven foam cell formation, and cardiac BCAA accumulation leading to myocardial hypertrophy. Epidemiological data: leucine/isoleucine HR 1.70/2.09 (PREDIMED); total BCAAs HR 1.13 (Women’s Health Study). **(F)** Consequence 3: neurotoxicity. BCAA/BCKA-mediated competitive inhibition of aromatic amino acid transport via LAT1 reduces tryptophan availability and serotonin synthesis; glutamate accumulation contributes to excitotoxicity. In MSUD, severe BCAA/BCKA accumulation causes metabolic acidosis and neurotoxicity. In hepatic encephalopathy, reduced Fischer ratio elevates AAA/BCAA, increasing HE risk—a clinical context in which BCAA supplementation is beneficial. BCAA, Branched-Chain Amino Acid; BCKA, Branched-Chain Keto Acid; TNF-α, Tumor Necrosis Factor-Alpha; IL-6, Interleukin-6; MCP-1, Monocyte Chemoattractant Protein-1; BCAT, Branched-Chain Amino Acid Transaminase; BCKDH, Branched-Chain Alpha-Keto Acid Dehydrogenase; BCKDK, Branched-Chain Alpha-Keto Acid Dehydrogenase Kinase; HMGB1, High Mobility Group Box 1; mTOR, Mechanistic Target of Rapamycin; mTORC1, Mechanistic Target of Rapamycin Complex 1; S6K1, Ribosomal Protein S6 Kinase Beta-1; IRS-1, Insulin Receptor Substrate 1; 3-HIB, 3-Hydroxyisobutyrate; T2DM, Type 2 Diabetes Mellitus; CVD, Cardiovascular Disease; HR, Hazard Ratio; eNOS, Endothelial Nitric Oxide Synthase; NO, Nitric Oxide; LAT1, L-Type Amino Acid Transporter 1; MSUD, Maple Syrup Urine Disease; HE, Hepatic Encephalopathy; AAA, Aromatic Amino Acid.

Parallel pathological mechanisms beyond mTORC1. While mTORC1/S6K1 hyperactivation is the most extensively characterized node, the pathological consequences of impaired BCAA catabolism involve at least three additional parallel mechanisms that operate independently of mTORC1. First, BCKAs—the immediate transamination products of BCAAs—accumulate when BCKDH flux is impaired and directly inhibit insulin-stimulated Akt phosphorylation, thereby impairing glucose uptake and mitochondrial oxygen consumption through mTORC1-independent routes (20, 29). Second, branched-chain acylcarnitines (BCACs), generated from incomplete BCAA oxidation, accumulate in skeletal muscle of insulin-resistant individuals, competitively inhibit fatty acid oxidation, and are associated with mitochondrial membrane dysfunction and oxidative stress (14, 30). Third, the resulting mitochondrial redox imbalance—characterized by elevated reactive oxygen species (ROS) production and impaired antioxidant capacity—activates NF-κB and NLRP3 inflammasome pathways, amplifying chronic inflammatory responses (31, 32). The integrated pathological network arising from these converging mechanisms is summarized in **Figures 2**, **3**. Where the available evidence is primarily epidemiological or associative, this review deliberately employs qualified language (e.g., “is associated with, “ “may contribute to”) rather than asserting direct causation.

#### 2.2.2 Interplay between insulin signaling and BCAA metabolism

**Figure 4:**
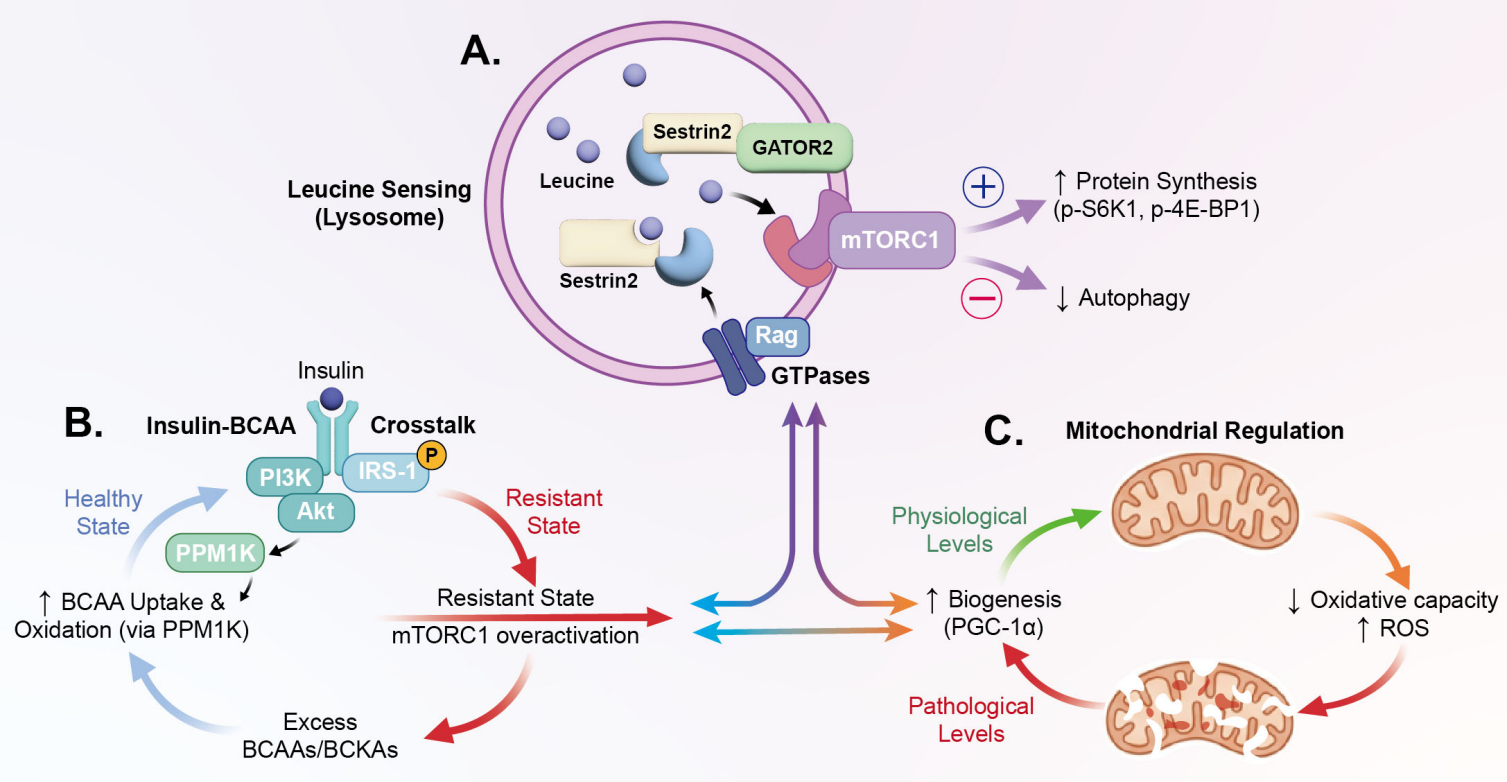
The tripartite signaling network: leucine sensing, insulin–BCAA crosstalk, and mitochondrial regulation This figure presents three interconnected intracellular signaling modules that collectively determine the context-dependent effects of BCAAs, emphasizing dose-dependence and metabolic-state-dependence of BCAA actions. **(A)** mTORC1 hub: leucine sensing. Leucine binding to Sestrin2 initiates the GATOR2–Rag GTPase signaling cascade, recruiting and activating mTORC1 at the lysosomal surface. Downstream phosphorylation of S6K1 and 4E-BP1 enhances mRNA translation and promotes protein synthesis while modulating autophagy flux. **(B)** Insulin–BCAA crosstalk loop. In the healthy state (blue arrows): insulin activates PPM1K via PI3K–Akt, promoting BCKDH activation, BCAA uptake, and oxidation, while maintaining normal IRS-1 tyrosine phosphorylation. In the insulin-resistant state (red arrows): excess BCAAs/BCKAs chronically hyperactivate mTORC1/S6K1, driving IRS-1 serine phosphorylation and impairing PI3K/Akt signaling—establishing a self-perpetuating vicious cycle of worsening BCAA accumulation and insulin resistance. **(C)** Mitochondrial regulation: dose-dependent effects. At physiological concentrations (green arrow): BCAAs stimulate PGC-1α-mediated mitochondrial biogenesis via AMPK, enhancing oxidative capacity. At pathological concentrations (red arrow): BCAAs suppress mitochondrial biogenesis gene expression, reduce oxidative capacity, increase ROS production, and impair mitochondrial quality control—establishing a bidirectional vicious cycle between BCAA accumulation and mitochondrial dysfunction. mTORC1, Mechanistic Target of Rapamycin Complex 1; GATOR1, GTPase-Activating Protein Toward Rags 1; GATOR2, GTPase-Activating Protein Toward Rags 2; Rag, Rag GTPase; S6K1, Ribosomal Protein S6 Kinase Beta-1; 4E-BP1, Eukaryotic Translation Initiation Factor 4E-Binding Protein 1; PI3K, Phosphoinositide 3-Kinase; Akt, Protein Kinase B; PPM1K, Protein Phosphatase, Mg2+/Mn2+ Dependent 1K; BCKDH, Branched-Chain Alpha-Keto Acid Dehydrogenase; BCAA, Branched-Chain Amino Acid; BCKA, Branched-Chain Keto Acid; IRS-1, Insulin Receptor Substrate 1; PGC-1α, Peroxisome Proliferator-Activated Receptor Gamma Coactivator 1-Alpha; AMPK, AMP-Activated Protein Kinase; ROS, Reactive Oxygen Species.

A complex bidirectional regulatory relationship exists between insulin signaling and BCAA metabolism. As illustrated in **Figure 4**, Under physiological conditions, insulin regulates BCAA metabolism through multiple mechanisms: suppressing whole-body protein breakdown to reduce endogenous BCAA release; promoting peripheral tissue BCAA uptake; and enhancing BCKDH activity via PPM1K activation, thereby accelerating BCAA oxidative catabolism (33).

However, in insulin-resistant states, this sophisticated regulatory network becomes disrupted. Insulin resistance leads to insensitivity to insulin’s anti-proteolytic effects, while peripheral tissue BCAA uptake and oxidative capacity decline, resulting in elevated plasma BCAA and BCKA levels (34). These accumulated metabolites further exacerbate insulin resistance through multiple mechanisms, particularly via excessive activation of the mTORC1/S6K1 pathway, inducing serine phosphorylation of insulin receptor substrate-1 (IRS-1) and thereby inhibiting insulin signal transduction (35). *In vitro* studies further confirm that BCKAs can directly interfere with insulin signaling, with mechanisms potentially involving mitochondrial dysfunction and reactive oxygen species generation (29).

#### 2.2.3 Mitochondrial function and BCAA oxidative capacity

As the sole site of BCAA oxidative metabolism, mitochondrial functional status directly determines catabolic efficiency. In metabolically healthy individuals, adequate mitochondrial oxidative phosphorylation capacity ensures efficient entry of BCAAs and their derivatives into the tricarboxylic acid cycle for complete oxidation. However, in patients with obesity, insulin resistance, and type 2 diabetes, skeletal muscle mitochondrial oxidative capacity is universally compromised, manifesting as reduced mitochondrial density and decreased respiratory chain complex activity (13). Notably, BCAAs exhibit dual regulatory characteristics on mitochondrial function. At physiological concentrations, BCAAs upregulate mitochondrial biogenesis-related gene expression through AMPK and PGC-1α activation, promoting mitochondrial function; conversely, abnormally elevated BCAA concentrations suppress mitochondrial biogenesis and oxidative gene expression (10). This dose-dependent effect further demonstrates the complexity of BCAA metabolic regulation (see **Figure 4**).

### 2.3 Pathological basis of metabolic dysregulation

#### 2.3.1 BCKDH activity reduction in obesity and insulin resistance

Substantial epidemiological evidence indicates that patients with obesity and insulin resistance exhibit significantly elevated plasma BCAA levels, closely associated with reduced tissue BCKDH activity (36). In genetic obesity mouse models, systemic suppression of BCAA catabolic gene expression can be observed, with elevated BCKDH phosphorylation (inactivation) levels, and these metabolic abnormalities often precede overt insulin resistance, suggesting that BCAA metabolic dysfunction may represent an early event in obesity-related metabolic disorders. Restoration of BCKDH activity has been proven to effectively ameliorate metabolic dysfunction. Pharmacological activation of BCKDH using small molecule BCKDK inhibitors (such as BT2) significantly reduces plasma BCAA and BCKA levels in obese mouse models and improves insulin sensitivity and glucose tolerance in preclinical settings (37). These studies provide experimental support for a contributory role of BCAA metabolic dysfunction in obesity-associated insulin resistance, though translation to human therapeutic application requires further validation of long-term safety and efficacy. (see **Figure 3**).

#### 2.3.2 Chronic inflammation-mediated suppression of BCAA metabolic enzymes

Chronic low-grade inflammation serves as an important link connecting obesity, insulin resistance, and BCAA metabolic dysregulation. In the obese state, adipose tissue undergoes macrophage infiltration and increased secretion of pro-inflammatory cytokines (such as TNF-α and IL-6), which suppress BCAA metabolic enzyme expression and activity through multiple mechanisms (38). For instance, research on diabetic retinopathy revealed that under conditions of hyperglycemia and BCAA accumulation, Müller cells exhibited decreased BCAT and BCKDH protein levels with elevated BCKDK expression, accompanied by increased levels of inflammatory factors such as TNF-α (39). Conversely, overexpression of metabolism-related proteins, such as HMGB1—a protein closely associated with cancer-related muscle wasting (40), cancer-related cardiac dysfunction (41), and skeletal muscle aging (42)—further impairs BCAA metabolism and promotes BCAA accumulation, establishing a vicious cycle of mutually reinforcing metabolic dysfunction and inflammation (43).

#### 2.3.3 BCAA accumulation due to mitochondrial dysfunction

A complex reciprocal relationship exists between mitochondrial dysfunction and BCAA accumulation. In age-related sarcopenia, recent mechanistic studies have unveiled an intriguing ‘mTOR paradox’: impaired mitochondrial BCAA catabolism in aged muscle leads to pathological BCAA accumulation, which excessively activates mTORC1. However, this activation not only fails to promote muscle growth but instead causes muscle mass and strength loss through autophagy suppression and mitochondrial dysfunction (44). This finding challenges the traditional notion that mTORC1 activation benefits muscle health, suggesting that under specific pathological conditions, ‘pathological mTORC1 hyperactivation’ fundamentally differs from ‘physiological mTORC1 activation.’ Pharmacological interventions enhancing BCKDH activity to promote BCAA catabolism have demonstrated protective effects in aged mouse models, providing novel strategic approaches for preventing and treating sarcopenia (45). In the cardiovascular disease domain, similar metabolic dysfunction has been observed. Heart failure patients and animal models exhibit elevated cardiac BCAA levels and increased BCKDH phosphorylation. Restoring cardiac BCAA catabolic capacity can ameliorate cardiac tissue remodeling and heart function, indicating that BCAA metabolic dysregulation plays critical roles in pathological processes across different tissues and organs (29).

The metabolism and regulation of BCAAs constitute a complex network system involving absorption, tissue distribution, enzymatic reactions, and signal transduction. From intestinal absorption through tissue-specific metabolism to the sophisticated regulation of the BCKDH rate-limiting step, each component is subject to fine-tuning by nutritional status, hormonal signals, and energy balance. Under physiological conditions, this system effectively maintains BCAA metabolic homeostasis. However, under pathological states such as obesity, chronic inflammation, and mitochondrial dysfunction, impaired BCAA catabolism leads to accumulation, participating in the pathogenesis and progression of insulin resistance, type 2 diabetes, cardiovascular disease, and sarcopenia through multiple mechanisms including excessive mTORC1 activation, insulin signaling interference, and mitochondrial stress induction. This distinctive regulatory relationship manifests as a ‘double-edged sword’ characteristic in disease development and progression. In **Figure 3**, we summarized the relationship between pathological drivers and multi-system damage.

## 3 The “angel” face of branched-chain amino acids: beneficial effects

### 3.1 Muscle protection and anabolic support

#### 3.1.1 Promotion of muscle protein synthesis

Leucine-mediated activation of the mTORC1 pathway: BCAAs, particularly leucine, are key regulators of muscle protein synthesis. Leucine activates the mTORC1 signaling pathway through the Sestrin2–GATOR2–Rag GTPase axis described in Section 2.2.1, promoting phosphorylation of S6K1 and 4E-BP1 and thereby stimulating mRNA translation (28, 46, 47). BCAA mixtures activate S6K1 more potently than leucine alone following exercise, and mixtures containing all essential amino acids produce the strongest response (28).

Attenuation of muscle protein degradation: BCAAs also suppress protein degradation. mTORC1 activation inhibits autophagy by phosphorylating ULK1, TFEB, and Beclin-1 (48). Leucine additionally suppresses ubiquitin-proteasome system activity (47).

Improvement of nitrogen balance: BCAA supplementation shifts net protein balance toward positive values during post-exercise recovery (49). Ingestion of 5.6 g BCAAs following resistance exercise increased myofibrillar protein synthesis rate by 22% and reduced phenylalanine release by approximately 6% (49).

#### 3.1.2 Prevention and management of muscle loss

##### 3.1.2.1 Sarcopenia in the elderly

Sarcopenia represents a common syndrome in older adults, characterized by progressive loss of skeletal muscle mass, decreased muscle strength, and reduced physical function (50). Research suggests that aging is accompanied by attenuated anabolic responses of muscle to nutritional stimuli, which may constitute an important mechanism underlying sarcopenia development. Nevertheless, elderly muscle maintains responsiveness to amino acid stimulation, particularly to BCAAs and essential amino acids (51, 52). This stimulatory effect is primarily attributed to the direct action of leucine on mRNA translation initiation, a mechanism that remains preserved in older individuals (52).

Multiple randomized controlled trials (RCTs) have evaluated the effects of BCAA supplementation on sarcopenia. A systematic review and meta-analysis including 35 RCTs demonstrated that BCAA supplementation improved muscle strength (standardized mean difference [SMD]: 0.35) and muscle mass (SMD: 0.25) in older adults, though improvements in physical function did not reach statistical significance (53). A 5-week BCAA supplementation study showed significant improvements in skeletal muscle mass index, gait speed, and grip strength in sarcopenic patients (54). However, these benefits disappeared 12 weeks after supplementation cessation, suggesting the need for continuous supplementation to maintain therapeutic effects.

##### 3.1.2.2 Cachexia-associated muscle wasting

Cancer cachexia represents a common complication in cancer patients, characterized by rapid skeletal muscle loss. Studies indicate that BCAAs, particularly leucine and valine, can significantly attenuate body weight loss and increase skeletal muscle wet weight in tumor-bearing mice through mechanisms involving enhanced protein synthesis and reduced protein degradation (55, 56). In the MAC16 tumor-induced cachexia model, daily oral administration of 1g/kg leucine or valine significantly suppressed body weight loss and muscle atrophy, with leucine additionally reducing protein degradation rate (55).

However, the efficacy of BCAAs in cancer cachexia treatment remains controversial. Some studies found that BCAA supplementation failed to significantly improve muscle oxidative stress, mitochondrial function, or cytokine levels in cachectic mice (43). Furthermore, the chemotherapy agent 5-fluorouracil exacerbated muscle loss in cachectic mice, and BCAA supplementation did not ameliorate this deterioration (43). These findings suggest that the effects of BCAAs in cachexia treatment may be influenced by multiple factors including tumor type, disease progression stage, and treatment regimen.

##### 3.1.2.3 Muscle protection during prolonged bed rest/weightlessness

Prolonged bed rest or weightlessness can lead to rapid muscle atrophy. Evidence suggests that BCAA supplementation may help mitigate disuse muscle atrophy through mechanisms involving mTORC1 pathway activation, maintenance of protein synthesis rates, and suppression of protein degradation pathway activation (57). However, clinical studies in this specific population remain limited, requiring further evidence.

#### 3.1.3 Summary of clinical evidence

##### 3.1.3.1 Meta-analysis results from randomized controlled trials

Multiple systematic reviews and meta-analyses have evaluated the effects of BCAA supplementation on muscle health. Meta-analyses focused on elderly sarcopenia have shown that BCAA supplementation improves muscle strength and mass, though results demonstrate considerable heterogeneity (53). Sources of heterogeneity may include supplementation dose, duration, study population characteristics, and whether combined with exercise training.

##### 3.1.3.2 Differential benefits across populations

The efficacy of BCAA supplementation varies across populations. Elderly individuals and sarcopenic patients may derive greater benefits from BCAA supplementation compared to healthy young adults. Additionally, individuals with malnutrition, chronic diseases (such as liver cirrhosis and chronic kidney disease), and those in postoperative recovery may also benefit from BCAA supplementation (58).

##### 3.1.3.3 Optimal dosage and duration considerations

Currently, no unified standard exists for optimal BCAA supplementation dose and duration. Doses used in studies range widely from 5g to over 30g daily. A reasonable starting dose is generally considered to be 0.2-0.4g/kg body weight per day (59). Regarding supplementation duration, most studies indicate that at least 4–12 weeks are required to observe significant effects. Furthermore, combining BCAAs with resistance exercise may produce superior synergistic effects.

### 3.2 Immune function support

#### 3.2.1 Substrate provision

##### 3.2.1.1 Substrate requirements for lymphocyte proliferation

Branched-chain amino acids play important roles in the immune system. Lymphocyte proliferation and functional maintenance require adequate amino acid supply, with BCAAs serving as important energy substrates and protein synthesis precursors (60). Research indicates that immune cells can produce decarboxylases and dehydrogenases to metabolize BCAAs, thereby improving lymphocyte growth, proliferation, and dendritic cell function.

##### 3.2.1.2 Amino acid sources for immunoglobulin synthesis

Immunoglobulin synthesis represents a key function of B lymphocytes, requiring sufficient amino acid supply. As essential amino acids, BCAAs constitute indispensable raw materials for immunoglobulin synthesis (61). In critical conditions such as sepsis, decreased immunoglobulin levels correlate with poor prognosis, and appropriate nutritional support including BCAA supplementation may help maintain immunoglobulin levels.

#### 3.2.2 Immune modulation

##### 3.2.2.1 Immune support in critically ill patients

Immune suppression represents a common complication in sepsis and other critical illnesses. Studies show that septic patients exhibit reduced peripheral blood lymphocyte counts with impaired T cell and B cell function (62, 63). BCAA supplementation as part of nutritional support may help improve nutritional status and immune function, though further clinical research is needed to confirm efficacy (64).

##### 3.2.2.2 Attenuation of excessive inflammatory responses

In systemic inflammatory response syndromes such as sepsis, excessive inflammatory responses can lead to tissue damage and organ dysfunction. Some studies suggest that BCAAs may attenuate excessive inflammation by modulating inflammatory mediator production. B lymphocytes play important roles in early sepsis immune responses, and BCAAs may improve immune responses by supporting B cell function (65).

#### 3.2.3 Summary of clinical evidence

##### 3.2.3.1 Prevention of postoperative infectious complications

Postoperative immune function may be suppressed, increasing infection risk. BCAAs as components of parenteral or enteral nutrition may help maintain postoperative immune function. Early studies suggest that BCAA supplementation may reduce postoperative infectious complication rates, though more high-quality clinical trials are needed to verify this effect.

##### 3.2.3.2 Nutritional support in septic patients

Septic patients commonly experience hypercatabolic states and immune dysfunction. Some prospective controlled trials have shown that BCAA supplementation can improve nutritional status and clinical outcomes in septic patients (64). However, the specific effects of BCAA supplementation may depend on supplementation dose, route (enteral or parenteral), and adequacy of baseline nutritional support.

##### 3.2.3.3 Adjuvant therapy in chronic infectious diseases

In chronic infectious diseases, persistent immune activation and inflammatory responses can lead to protein-energy malnutrition. BCAA supplementation as part of nutritional support strategies may help maintain immune function and improve nutritional status, though individualized assessment based on specific disease types is necessary.

### 3.3 Hepatic protective effects

#### 3.3.1 Liver cirrhosis and hepatic encephalopathy

##### 3.3.1.1 Correction of amino acid imbalance

Liver cirrhosis is characterized by decreased BCAA levels and elevated aromatic amino acid (AAA) levels, resulting in a reduced Fischer ratio (66, 67). This imbalance contributes to hepatic encephalopathy (HE). BCAA supplementation corrects the Fischer ratio and improves HE symptoms by competitively inhibiting AAA transport across the blood-brain barrier (68).

A Cochrane review of 16 RCTs (n = 827) found that BCAA supplementation significantly improved HE manifestations (RR 0.73, 95% CI 0.61–0.88; NNT = 5), with consistent results in low-bias-risk trials (RR 0.71, 95% CI 0.52–0.96) (68). Oral BCAAs also improved HE manifestations across a meta-analysis of randomized trials (risk ratio 1.71, 95% CI 1.17–2.51), with stronger effects in overt HE (risk ratio 3.26, 95% CI 1.47–7.22) (69).

##### 3.3.1.2 Ammonia modulation

When hepatic detoxification fails, skeletal muscle becomes the primary ammonia-clearing organ. BCAAs are catabolized in skeletal muscle and participate in ammonia metabolism (24). Intravenous BCAA administration may paradoxically increase ammonia levels; the therapeutic benefit of BCAAs in HE thus likely derives primarily from Fischer ratio correction rather than direct ammonia reduction (67).

##### 3.3.1.3 Hepatic synthetic function and regeneration

Branched-chain amino acids may promote hepatocyte proliferation and support liver regeneration through mTORC1 activation (see Section 2.2.1) (67). Long-term supplementation improves hepatic protein synthesis and nutritional parameters in cirrhotic patients (70). Some evidence suggests BCAA supplementation may slow fibrosis progression and reduce hepatocellular carcinoma risk, though larger trials are needed (67, 70).

#### 3.3.2 Clinical evidence and guideline recommendations

The therapeutic benefits of BCAA supplementation in advanced cirrhosis are supported by a robust body of randomized controlled trial evidence and major clinical guidelines, representing one of the most well-established “angel” applications of BCAAs in chronic disease management.

In a multicenter double-blind RCT, long-term oral BCAA supplementation significantly reduced the composite risk of death, liver transplantation, and disease progression in patients with advanced cirrhosis compared with lactalbumin or maltodextrin controls, with event-free survival significantly improved in the BCAA group (9). Among cirrhotic patients with hypoalbuminemia, BCAA granule supplementation (12 g/day for 2 years) significantly improved serum albumin levels, reduced the incidence of ascites and edema, and improved health-related quality of life; benefits were most pronounced in patients with lower baseline albumin and Fischer ratio, identifying the subgroup most likely to derive clinical benefit (30).

At the highest level of evidence, a Cochrane systematic review and meta-analysis incorporating 16 RCTs (n = 827) confirmed that oral BCAA supplementation significantly reduced the risk of hepatic encephalopathy (risk ratio 0.73, 95% CI 0.61–0.88; number needed to treat = 5) and improved overall clinical manifestations in cirrhotic patients, with the beneficial effect remaining robust in trials with low risk of bias (risk ratio 0.71, 95% CI 0.52–0.96) (66). Although a definitive survival benefit has not yet been established, consistent improvements in hepatic encephalopathy, nutritional parameters, and event-free survival across multiple trials provide compelling evidence for clinical application.

These findings are reflected in international clinical nutrition guidelines. Long-term oral BCAA supplementation is formally recommended for decompensated cirrhotic patients presenting with low Fischer ratio, sarcopenia, or protein-energy malnutrition, typically as leucine-enriched formulations at 12–14 g/day for a minimum of six months, targeting Fischer ratio correction, nutritional rehabilitation, hepatic encephalopathy risk reduction, and skeletal muscle ammonia clearance support (71). This constitutes a Grade A recommendation based on consistent RCT evidence.

Taken together, the available evidence positions BCAA supplementation as a disease-modifying nutritional intervention in advanced cirrhosis, with benefits operating through the three converging mechanisms outlined in Section 3.3.2: Fischer ratio correction, skeletal muscle ammonia clearance support, and mTORC1-mediated promotion of hepatocyte proliferation and protein synthesis. Critically, this therapeutic context—decompensated cirrhosis with documented malnutrition and reduced Fischer ratio—is mechanistically and clinically distinct from early MASLD or metabolic syndrome, where elevated circulating BCAAs reflect impaired catabolic disposal rather than insufficient intake, and supplementation is not recommended (see Section 6.3 for disease-stage-stratified clinical recommendations).

#### 3.3.3 Summary of mechanistic basis

The hepatoprotective effects of BCAAs in cirrhosis operate through three converging mechanisms: (1) correction of the Fischer ratio and competitive inhibition of AAA brain entry; (2) support of skeletal muscle ammonia clearance; and (3) mTORC1-mediated promotion of hepatocyte proliferation and protein synthesis. These mechanisms are disease-stage dependent and are discussed in the clinical context in Section 6.3.

### 3.4 Other beneficial effects

#### 3.4.1 Improvement of wound healing

Branched-chain amino acids may play important roles in tissue repair and wound healing. Research indicates that BCAAs participate in promoting protein synthesis and cell proliferation, processes crucial for wound healing (60, 69). In cachectic patients, malnutrition and muscle wasting may impair wound healing, and BCAA supplementation as part of nutritional support may help ameliorate this condition (69). However, direct effects of BCAAs on wound healing require further clinical research confirmation.

#### 3.4.2 Support of nervous system function

Branched-chain amino acids are closely related to brain tryptophan and serotonin levels. BCAAs compete with aromatic amino acids for transport across the blood-brain barrier; therefore, changes in BCAA levels may affect neurotransmitter balance in the central nervous system (70). In HE, this mechanism may represent an important pathway through which BCAAs exert therapeutic effects. Additionally, BCAAs may play roles in maintaining mental strength and promoting neuroprotection (60).

#### 3.4.3 Antioxidant and anti-stress effects

Some studies suggest that BCAAs may possess antioxidant properties. In cancer cachexia animal models, although BCAA supplementation did not significantly improve muscle oxidative stress markers, it may exert anti-stress effects in certain situations by supporting mitochondrial function and cellular energy metabolism (43, 57). Furthermore, BCAAs may reduce stress-induced muscle catabolism by improving protein synthesis and reducing protein degradation.

Branched-chain amino acids play important roles in promoting muscle protein synthesis, preventing and treating muscle loss, supporting immune function, and protecting hepatic function. Substantial clinical research confirms that BCAA supplementation improves muscle strength and mass in elderly individuals, ameliorates HE symptoms, and supports immune function in critically ill patients. However, clinical application of BCAAs requires individualized assessment, considering patient-specific disease states, nutritional status, and therapeutic goals. Future research should focus on high-quality RCTs to clarify optimal populations, doses, and durations for BCAA application, and explore synergistic effects of BCAAs with other therapeutic interventions.

## 4 The “dark side” of branched-chain amino acids

### 4.1 Insulin resistance and glucose metabolic disorders

#### 4.1.1 Association between branched-chain amino acids and insulin resistance

Accumulating epidemiological evidence has demonstrated a strong association between elevated plasma BCAAs levels and insulin resistance (IR). In a pioneering metabolomics study published in 2009, Newgard et al. revealed that BCAAs and their related metabolites exhibited the strongest correlation with insulin sensitivity in obese and insulin-resistant individuals, surpassing even many lipid species (10). Subsequently, Newgard further elucidated the interplay between lipids and BCAAs in the development of insulin resistance in 2012 (72). In a comprehensive systematic review published in 2014, Lynch and Adams demonstrated that fasting circulating BCAA concentrations were universally elevated in obese populations—reflecting impaired catabolic disposal rather than excess dietary intake—and positively correlated with the future risk of developing type 2 diabetes (T2D) (14).

Clinical cohort studies have provided robust evidence supporting this association. A follow-up study of women with gestational diabetes mellitus (GDM) revealed that patients who developed T2D after 6 years exhibited significantly elevated plasma BCAA levels, which were closely associated with insulin resistance indices (73). The PREVEND cohort study, encompassing 5, 791 participants with a median follow-up of 7.3 years, demonstrated that higher BCAA levels were independently associated with T2D incidence (hazard ratio [HR] = 1.19), and the association between fatty liver and T2D was partially mediated by elevated BCAAs, with a mediation proportion of 19.6% (74).

Regarding causality, Zhou et al. discovered in 2019 through integrated pathway analysis that systemic inhibition of BCAA catabolic enzymes accompanied by BCAA accumulation in genetically obese mice could be ameliorated by the BCKDK inhibitor BT2, which restored BCAA catabolism and significantly improved insulin resistance (36). Zhao et al., in a series of studies published in 2020 and 2024, further demonstrated that a single BCAA infusion acutely elevated blood glucose and insulin levels, while continuous infusion impaired whole-body insulin sensitivity; notably, BT2 treatment improved glucose tolerance in high-fat diet-fed mice (75, 76).

**Figure 5:**
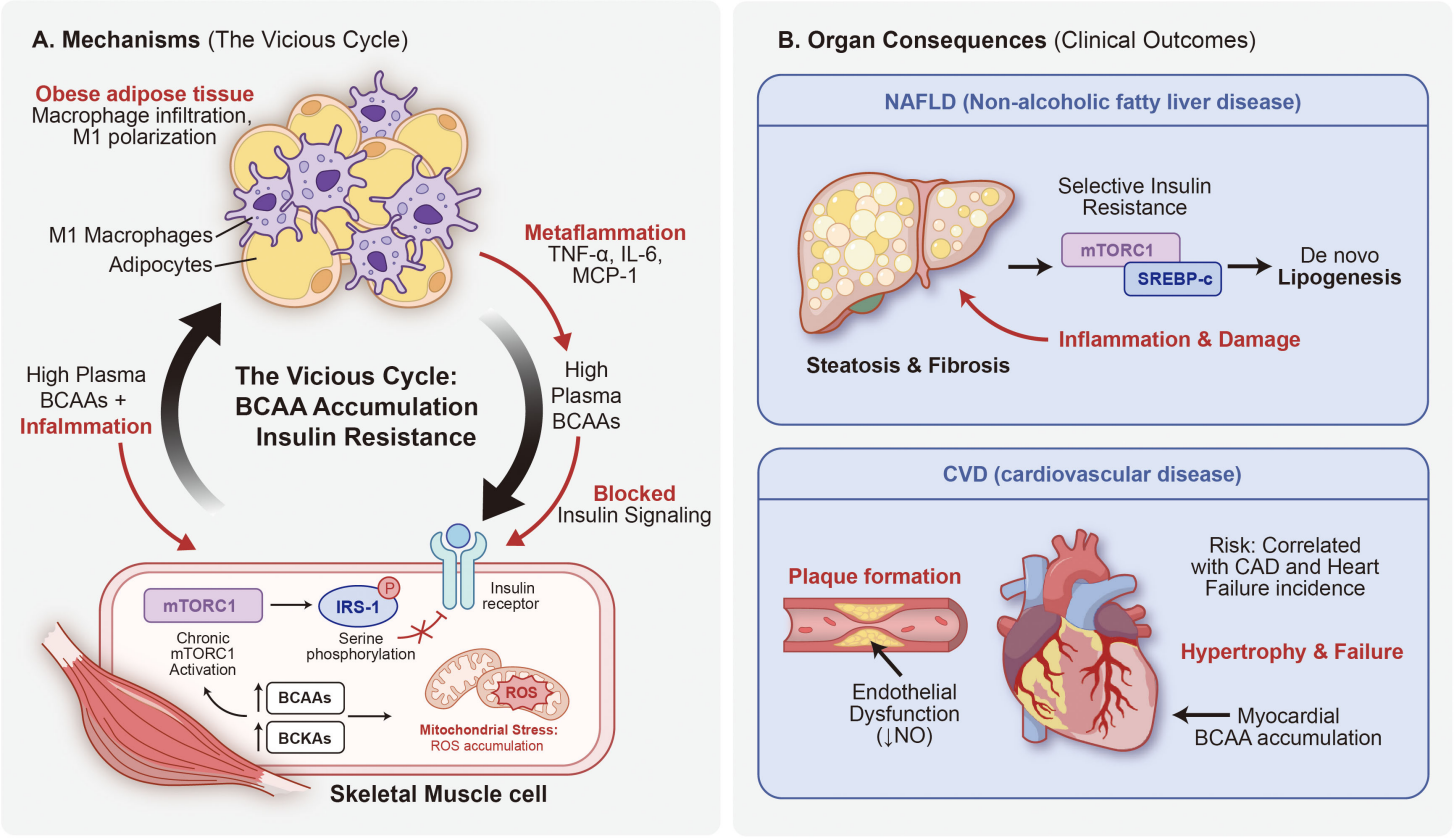
The Metabolic Vicious Cycle: Insulin Resistance, Inflammation, and Organ Damage. This figure integrates mechanistic pathways with clinical consequences, presenting a dual-panel format that bridges molecular events with organ-level pathology. **(A)** Left panel - Mechanisms: The mechanistic loop illustrates the bidirectional crosstalk between adipose tissue and skeletal muscle. Adipose tissue serves as the inflammatory source, characterized by macrophage infiltration, M1 polarization, and release of pro-inflammatory mediators (TNF-α, IL-6, MCP-1), establishing the metaflammatory milieu that suppresses BCAA catabolic enzymes. Skeletal muscle serves as the site of insulin resistance development, where elevated plasma BCAAs combined with chronic inflammation trigger sustained mTORC1 activation, IRS-1 serine phosphorylation, and mitochondrial stress with ROS accumulation from BCKAs. This establishes a self-perpetuating vicious cycle between BCAA accumulation and insulin resistance. **(B)** Right panel - Organ consequences: The clinical manifestations include hepatic and cardiovascular complications. Liver (MASLD): Selective insulin resistance leads to mTORC1-mediated SREBP-1c activation, promoting *de novo* lipogenesis and resulting in hepatic steatosis and fibrosis. Cardiovascular system: Vascular consequences include endothelial dysfunction (characterized by reduced nitric oxide [NO] bioavailability) and atherosclerotic plaque formation. Cardiac consequences include myocardial BCAA accumulation leading to hypertrophy and eventual heart failure. Epidemiological studies confirm correlations between elevated BCAAs and increased incidence of coronary artery disease (CAD) and heart failure. BCAA, Branched-Chain Amino Acid; TNF-α, Tumor Necrosis Factor-Alpha; IL-6, Interleukin-6; MCP-1, Monocyte Chemoattractant Protein-1; mTORC1, Mechanistic Target of Rapamycin Complex 1; IRS-1, Insulin Receptor Substrate 1; ROS, Reactive Oxygen Species; BCKA, Branched-Chain Keto Acid; MASLD, Metabolic dysfunction-associated steatotic liver disease; SREBP-1c, Sterol Regulatory Element-Binding Protein 1c; NO, Nitric Oxide; CAD, Coronary Artery Disease.

It is noteworthy that the effects of BCAAs on insulin sensitivity exhibit a concentration-dependent pattern: beneficial at physiological levels but deleterious at pathological concentrations (14, 22). Blair et al.’s 2023 study suggested that elevated BCAAs might serve as biomarkers rather than direct causative factors of insulin resistance (77), whereas Shah et al.’s acute intervention study provided compelling evidence for direct pathogenic effects (76). This controversy warrants further investigation. This metabolic vicious cycle is clearly illustrated in **Figure 5**.

#### 4.1.2 Mechanistic insights

The mTORC1/S6K1 pathway is one major mechanistic node linking chronically elevated circulating BCAAs—arising from impaired catabolic clearance—to insulin resistance, operating in parallel with BCKA-mediated direct inhibition of Akt signaling and BCAC-driven mitochondrial dysfunction, as described in Section 2.2.1. Under nutrient-excess conditions, sustained leucine elevation drives chronic mTORC1 activation, leading to IRS-1 serine phosphorylation and impaired insulin signaling (14). Dysregulated BCAA catabolism results in branched-chain acylcarnitine (BCAC) accumulation. These intermediates inhibit fatty acid oxidation, induce mitochondrial dysfunction, and promote oxidative stress (14, 72).BCKAs further suppress insulin signaling through mTORC1 activation (36).

Mitochondrial dysfunction and BCAA accumulation form a bidirectional vicious cycle: impaired oxidative capacity reduces BCAA catabolism, while accumulated intermediates exacerbate mitochondrial stress and ROS generation (3, 9). Elevated BCAAs also link to chronic inflammation, with pro-inflammatory cytokines promoting IRS-1 serine phosphorylation (4).

#### 4.1.3 Clinical evidence and intervention strategies

Multiple clinical studies have reported significantly elevated plasma BCAA levels in patients with T2D. The GDM follow-up study demonstrated markedly higher baseline BCAA levels in patients who progressed to T2D (73). The PREVEND cohort study further confirmed the independence of this association and its mediating role (74).

Exercise intervention may represent an effective approach to modulate BCAA metabolism. Research has shown that exercise enhances mitochondrial oxidation of BCAAs, and BCAA supplementation in athletes typically does not induce insulin resistance (78). Zhou et al.’s study demonstrated that dietary BCAA restriction improved insulin sensitivity in obese mice, whereas increased BCAA intake promoted insulin resistance (36), suggesting potential therapeutic value of dietary modification.

### 4.2 Promotion of chronic inflammation

#### 4.2.1 Inflammatory activation mechanisms

Nuclear factor-κB (NF-κB) and NOD-like receptor family pyrin domain-containing 3 (NLRP3) inflammasome represent key pathways mediating chronic inflammation. NF-κB serves as the principal transcription factor for inflammatory responses, regulating the expression of pro-inflammatory cytokines, chemokines, and adhesion molecules (31, 32). NLRP3 inflammasome activation requires two signals: a priming signal (activating the NF-κB pathway) and an activation signal (mediated through mechanisms including potassium efflux, mitochondrial dysfunction, and ROS generation) (79, 80).

Interestingly, NF-κB exhibits a dual role in inflammation regulation, functioning as both an initiator and a negative regulator. Greten et al. first discovered in 2007 that NF-κB could act as a negative regulator of interleukin-1β (IL-1β) secretion (81). Zhong et al. further confirmed in 2016 that NF-κB suppresses inflammasome activation by inducing p62 accumulation and eliminating damaged mitochondria through mitophagy (82).

Although direct evidence of BCAA-mediated activation of these pathways is lacking, mitochondrial dysfunction, ROS production, and metabolic intermediate accumulation resulting from BCAA metabolic dysregulation can all function as damage-associated molecular patterns (DAMPs) to activate inflammatory pathways. Chronic inflammation is characterized by sustained release of pro-inflammatory cytokines including interleukin-6 (IL-6), tumor necrosis factor-α (TNF-α), and monocyte chemoattractant protein-1 (MCP-1). These cytokines induce insulin resistance by activating stress kinases that promote IRS-1 serine phosphorylation (14).

In obesity and metabolic syndrome, adipose tissue macrophages predominantly polarize toward the pro-inflammatory M1 phenotype, releasing pro-inflammatory mediators that exacerbate local and systemic inflammation (83). Hotamisligil systematically elaborated in 2017 on the complex interrelationships among inflammation, metaflammation, and immunometabolic disorders (84). Metabolic dysregulation induced by aberrant BCAA metabolism may promote M1 polarization and adipose tissue inflammation through multiple mechanisms.

#### 4.2.2 Vicious cycle with chronic diseases

A vicious cycle exists among obesity, inflammation, and BCAA metabolic dysfunction (14, 73, 74). In the obese state, decreased expression of BCAA catabolic enzymes in adipose tissue leads to BCAA elevation; elevated BCAAs promote lipid accumulation through mTORC1 activation; insulin resistance further increases BCAA appearance rates. Concurrently, inflammation suppresses BCAA catabolic enzyme expression, while BCAA metabolic dysregulation activates inflammatory pathways.

A Chinese population study demonstrated that BCAA levels positively correlated with metabolic syndrome and its components. Subjects in the highest quartile of BCAA levels exhibited a 2.17-fold increased risk of metabolic syndrome and were independently associated with high cardiovascular risk scores (85). Multi-system inflammation in metabolic syndrome patients manifests as immune cell infiltration and pro-inflammatory mediator release in multiple organs, not only exacerbating insulin resistance but also promoting complications including atherosclerosis and MASLD.

Inflammation plays a central role in atherosclerosis development and progression (84). BCAAs promote vascular inflammation and atherosclerosis through multiple mechanisms, including insulin resistance induction, endothelial cell inflammatory response activation, and oxidative stress promotion.

#### 4.2.3 Clinical and experimental evidence

Multiple clinical studies have reported positive correlations between BCAA levels and inflammatory markers. The GDM follow-up study demonstrated that patients progressing to T2D exhibited not only elevated BCAA levels but also significantly increased C-reactive protein (CRP), triglycerides, and free fatty acids (73). In obese adolescents, BCAA levels correlated with inflammatory markers and metabolic abnormalities (86).

Zhou et al.’s study showed that increased BCAA intake promoted insulin resistance in ob/ob mice, an effect associated with inflammatory activation (36). Interventions improving inflammatory status, such as weight reduction and exercise, can restore BCAA catabolic enzyme activity and reduce circulating BCAA levels (78), suggesting bidirectional regulation between inflammation and BCAA metabolism.

### 4.3 Cardiovascular disease risk

#### 4.3.1 Epidemiological associations

Multiple large-scale prospective cohort studies have revealed associations between elevated fasting circulating BCAA concentrations and cardiovascular disease (CVD) risk. In the PREDIMED study published in 2016, Guasch-Ferré et al. found that the highest quartile concentrations of leucine and isoleucine were associated with increased CVD risk, with hazard ratios of 1.70 and 2.09, respectively (87). Tobias et al., in a study of 27, 041 women with a mean follow-up of 18.6 years published in 2018, found that total BCAAs positively correlated with CVD (HR = 1.13), with effect sizes comparable to low-density lipoprotein cholesterol (LDL-C) (88).

A meta-analysis by Wang et al. published in 2022, incorporating 43, 895 participants, demonstrated that elevated circulating isoleucine levels were independently associated with increased CVD risk (89). In the Women’s Health Study, incorporating BCAAs into traditional risk factor models increased the area under the receiver operating characteristic curve (AUC) from 0.718 to 0.721, with predictive value comparable to LDL-C (88).

The association between BCAAs and CVD varies across different populations. Sex, ethnicity, and metabolic status may all influence this relationship. A 2021 genetic study by Hu et al. demonstrated that the protein phosphatase 1K (PPM1K) gene polymorphism rs1440581 CC genotype was associated with elevated serum BCAAs and served as a superior marker for CVD and MASLD risk (90).

#### 4.3.2 Pathological mechanisms

Elevated BCAAs promote cardiovascular disease through multiple mechanisms: induction of insulin resistance and metabolic syndrome; promotion of vascular smooth muscle cell proliferation and macrophage foam-cell transformation via mTORC1 activation (see Section 2.2.1); and effects of BCAA metabolites such as 3-HIB on vascular lipid transport (91).

Endothelial dysfunction arises from impaired eNOS activation, oxidative stress-induced endothelial damage, and mTORC1-driven inflammatory responses (91). In heart failure, downregulation of BCAA catabolic enzymes leads to cardiac BCAA accumulation, potentially impairing cardiac metabolism through mTORC1 activation (91).

#### 4.3.3 Clinical implications and intervention strategies

Branched-chain amino acids may serve as biomarkers for CVD risk stratification. Reducing BCAA levels could potentially become a strategy for CVD prevention, although this must be balanced against the need to maintain muscle mass. Lifestyle interventions, particularly exercise training, may represent an ideal approach to modulate BCAA metabolism (78). While the Mediterranean diet has limited effects on BCAA levels, it may counteract the harmful effects of BCAAs through other mechanisms (87).

BCAA supplementation strategies in heart failure patients remain controversial. Although supplementation may improve nutritional status and exercise capacity, it could also exacerbate cardiac metabolic burden (91). BCAA supplementation in heart failure patients requires individualized assessment, comprehensively considering nutritional status, cardiac function, and metabolic phenotype.

The value of BCAAs as prognostic biomarkers for CVD is being explored. In T2D patients, BCAAs are associated with cardiovascular complications and mortality risk (89). Combining BCAAs with other biomarkers may provide more comprehensive risk assessment.

### 4.4 Metabolic dysfunction-associated steatotic liver disease: pathological mechanisms in non-cirrhotic disease

#### 4.4.1 Bidirectional relationship between BCAAs and MASLD in non-cirrhotic disease

The pathological mechanisms described in this section pertain specifically to early and non-cirrhotic MASLD/MASH, a stage at which fasting circulating BCAA concentrations are characteristically elevated as a consequence of impaired catabolic disposal. This context is mechanistically and clinically distinct from advanced MASH-related cirrhosis, in which circulating BCAAs decline and supplementation becomes indicated; the latter is addressed in Section 6.3.2. Non-cirrhotic MASLD patients commonly exhibit aberrant BCAA metabolism, characterised by elevated rather than depleted fasting circulating BCAA concentrations. McCormack et al.’s 2017 study of 78 obese adolescents demonstrated that MASLD patients exhibited significantly elevated plasma valine and isoleucine levels, independent of obesity and insulin resistance, with baseline valine levels predicting increased liver fat content during follow-up (86).

The PREVEND cohort study confirmed that fasting circulating BCAA concentrations positively correlated with fatty liver index, independent of the homeostatic model assessment of insulin resistance (HOMA-IR). Mediation analysis indicated that the association between MASLD and T2D was partially mediated by elevated BCAAs, with a mediation proportion of 19.6% (74), suggesting that elevated circulating BCAAs—reflecting impaired catabolic disposal at the non-cirrhotic disease stage—represent a critical metabolic link between MASLD and T2D.

The area and heterogeneity of hepatic lipid droplets correlated with plasma BCAA levels (92). BCAAs may participate in disease progression from simple steatosis to metabolic dysfunction-associated steatohepatitis (MASH, formerly NASH) through multiple mechanisms, though this pro-disease role is specific to the non-cirrhotic stage in which catabolic impairment—rather than deficiency—drives BCAA accumulation, including promotion of lipogenesis, induction of mitochondrial dysfunction, and activation of inflammatory pathways.

#### 4.4.2 Mechanistic insights in non-cirrhotic MASLD/MASH

In non-cirrhotic MASLD/MASH, chronically elevated circulating BCAAs promote hepatic lipogenesis through several mechanisms. Chronically elevated circulating BCAAs, driven by impaired catabolic disposal, induce selective insulin resistance, increasing adipose lipolysis and hepatic lipogenesis. mTORC1 activation (see Section 2.2.1) upregulates SREBP-1c and fatty acid synthase expression (75, 76). BCAA metabolic intermediates may also provide carbon sources for fatty acid synthesis.

BCAA catabolic intermediate accumulation competitively inhibits fatty acid oxidation and impairs mitochondrial metabolism in MASLD (93). Downregulation of PPARα is associated with decreased BCAA catabolic enzyme expression in hepatocellular carcinoma (94). BCAA infusion reduces insulin-stimulated pAkt in the liver by approximately 30%, indicating hepatic insulin resistance (75). The valine metabolite 3-HIB promotes trans-endothelial fatty acid transport and ectopic lipid accumulation within skeletal muscle, contributing to local lipotoxicity and insulin resistance (23, 91).

#### 4.4.3 Therapeutic implications in non-cirrhotic MASLD/MASH

In non-cirrhotic MASLD/MASH with elevated fasting circulating BCAAs, dietary BCAA restriction or avoidance of supplementation may improve metabolic outcomes. Animal studies have demonstrated that protein restriction improves metabolic health (36). Galarregui et al.’s 2021 human study suggested that optimizing amino acid composition may benefit liver status in MASLD patients (95). However, nutritional requirements must be balanced to avoid muscle wasting and malnutrition.

Pharmacological therapies targeting BCAA metabolism represent a theoretically appealing but predominantly preclinical avenue. The BCKDK inhibitor BT2 improves insulin sensitivity by promoting BCAA catabolism in animal models (36), though human safety data and optimal dosing parameters remain undefined. Other potential targets under investigation include branched-chain amino acid transaminase (BCAT), BCAA transporters, and related transcription factors; all require prospective clinical evaluation before therapeutic recommendations can be made.

Lifestyle intervention remains the cornerstone of MASLD treatment. Weight reduction of 5-10% can significantly improve MASLD (95, 96). Shi et al.’s 2021 study demonstrated that exercise training enhances BCAA oxidation, reduces circulating BCAA levels, and correlates BCAA changes with liver fat improv MASLD ement (97). The benefits of combined dietary, exercise, and behavioral interventions may exceed those of single interventions.

It should be emphasised that these therapeutic considerations apply exclusively to non-cirrhotic MASLD/MASH. In patients with advanced MASH-related decompensated cirrhosis, the metabolic profile reverses—circulating BCAAs decline, Fischer ratio falls, and oral BCAA supplementation is supported by Grade A guideline recommendations; this distinct clinical scenario is addressed in Section 6.3.2.

### 4.5 Potential pro-tumorigenic effects

#### 4.5.1 Tumor cell dependence on branched-chain amino acids

Rapidly proliferating tumor cells require substantial BCAA support (98, 99). BCAAs serve as building blocks for protein synthesis, provide nitrogen sources for nucleotide and non-essential amino acid biosynthesis, and contribute to energy production through oxidation. Li and Zhang systematically elaborated in 2016 on how tumor cells reprogram glucose, fatty acid, and amino acid metabolism to support cancer progression (100).

Tumor cells exhibit significant BCAA metabolic reprogramming, with heterogeneity across tumor types (60, 98, 99). Ananieva and Wilkinson’s 2018 review indicated that BCAA catabolic enzyme expression is suppressed in hepatocellular carcinoma and multiple other tumors (99). Ericksen et al.’s 2019 study demonstrated that the degree of enzyme inhibition strongly correlated with tumor aggressiveness and served as an independent prognostic predictor (94). Inhibition mechanisms include genomic deletions and altered transcription factor (e.g., PPARα) function.

Conversely, BCAT1 expression is upregulated in certain tumors, potentially promoting tumor growth through glutamate generation, provision of nitrogen sources for nucleotide synthesis, and mTORC1 signaling activation (60). Vettore et al.’s 2020 review discussed new insights into amino acid metabolism in cancer (98).

The mTOR pathway plays a central role in tumorigenesis and progression (94, 98, 100). BCAAs activate mTORC1 through the Sestrin2-GATOR-Rag GTPase axis. Downregulation of BCAA catabolic enzymes in tumors leads to BCAA accumulation, providing sustained mTORC1 activation signals. Ericksen et al. demonstrated that modulating BCAA accumulation can regulate cancer cell proliferation and tumor burden (94).

#### 4.5.2 Evidence for BCAA-promoted tumorigenesis

*In vitro* experiments demonstrated decreased BCAA catabolic enzyme expression in tumorigenic hepatocellular carcinoma (HCC) cell lines. Knockout or inhibition of BCAA catabolism reduced cancer cell proliferation rates and mTORC1 activity. BCAA supplementation promoted cancer cell proliferation, which could be blocked by mTOR inhibitors (94).

Animal model studies provided more direct evidence. In diethylnitrosamine (DEN)-induced HCC models, high-BCAA diets promoted tumor development, while BT2 treatment significantly improved tumor burden and overall survival. In xenograft models, BT2 treatment also significantly inhibited tumor growth (94).

**Figure 6:**
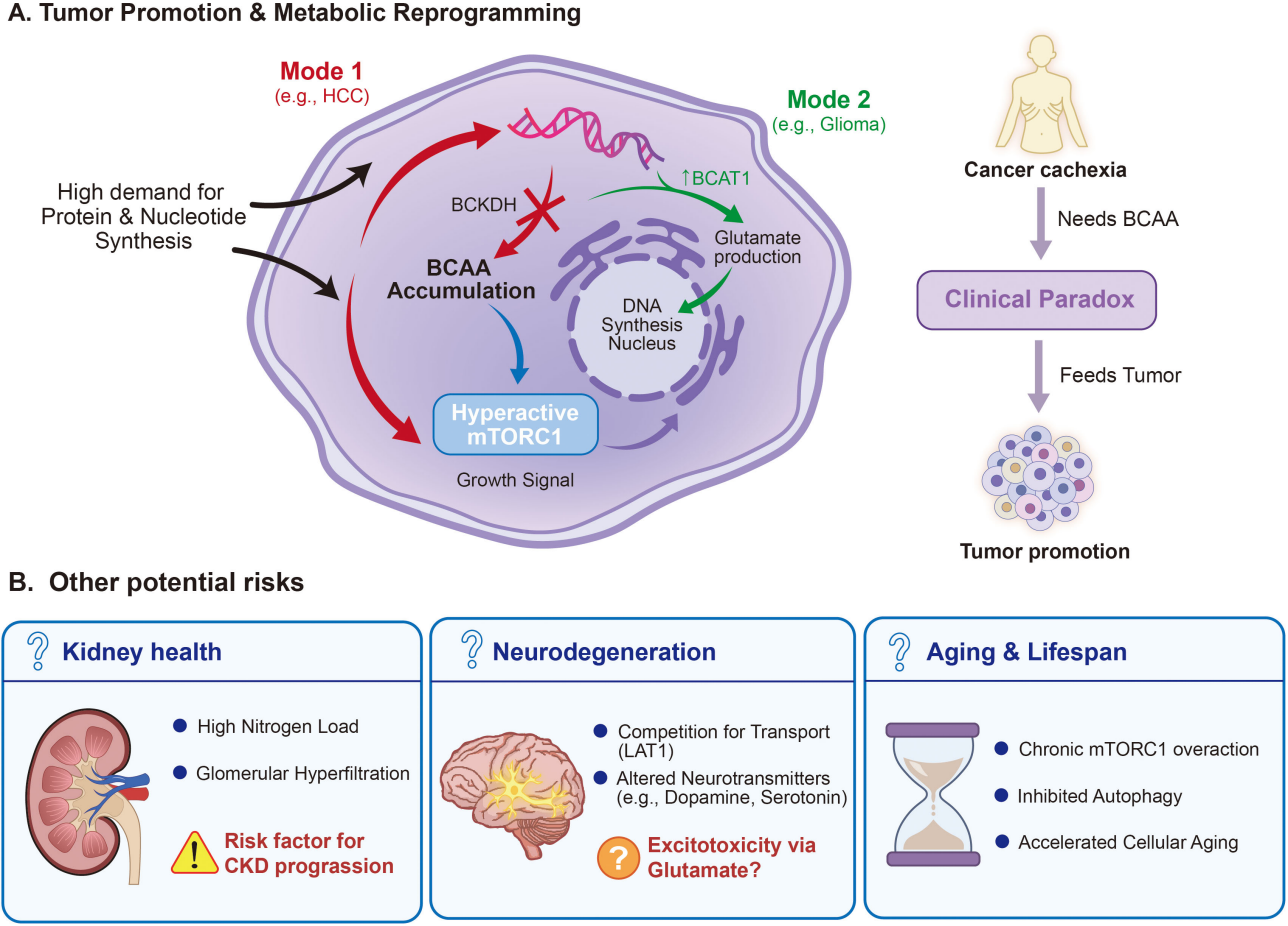
BCAA Dysregulation in Cancer and Emerging Risks. This figure addresses the dual role of BCAAs in tumorigenesis and presents emerging areas of concern, highlighting the “double-edged sword” nature of these amino acids in cancer metabolism. **(A)** Top panel - Tumor promotion mechanisms: Rapidly proliferating tumor cells exhibit metabolic reprogramming with high demand for protein and nucleotide synthesis substrates. Two distinct patterns emerge: Mode 1 (exemplified by hepatocellular carcinoma [HCC]): Downregulation of BCKDH leads to intracellular BCAA accumulation, providing sustained mTORC1 activation that serves as a growth signal. Mode 2 (exemplified by glioma): Upregulation of BCAT1 promotes glutamate production and provides nitrogen sources for DNA synthesis. This heterogeneity creates a clinical paradox: cachexic cancer patients require nutritional support including BCAAs, yet supplementation may potentially fuel tumor growth. **(B)** Bottom panel - Other potential risks: Three emerging areas of concern are presented. Kidney health: High nitrogen load from excessive BCAA intake may cause glomerular hyperfiltration and represent a risk factor for chronic kidney disease (CKD) progression. Neurodegeneration: BCAAs compete with aromatic amino acids for transport across the blood-brain barrier via LAT1, potentially altering neurotransmitter synthesis (dopamine, serotonin) and contributing to excitotoxicity through glutamate accumulation (though this remains controversial). Aging and lifespan: Chronic mTORC1 overactivation by elevated BCAAs inhibits autophagy, potentially accelerating cellular aging. Visual elements (scales or question marks) indicate the ongoing scientific uncertainty regarding these emerging risks. BCAA, Branched-Chain Amino Acid; HCC, Hepatocellular Carcinoma; BCKDH, Branched-Chain Alpha-Keto Acid Dehydrogenase; mTORC1, Mechanistic Target of Rapamycin Complex 1; BCAT1, Branched-Chain Amino Acid Transaminase 1; DNA, Deoxyribonucleic Acid; CKD, Chronic Kidney Disease; LAT1, L-Type Amino Acid Transporter 1.

Clinical studies demonstrated that BCAA catabolic enzyme expression levels in HCC patient tumor tissues correlated with tumor aggressiveness and prognosis. Human dietary cohort studies showed that replacing carbohydrates or fats with BCAAs was associated with a dose-dependent increase in cancer mortality risk (94). High BCAT1 expression typically correlates with more aggressive tumor growth (60, 99). Xu et al.’s 2023 review discussed the prospects for therapeutic interventions targeting BCAA catabolism (60). **Figure 6** synthesizes the aforementioned mechanisms, highlighting the central role of BCAA dysregulation in cancer metabolism and its associated emerging risks.

#### 4.5.3 Clinical dilemma

Cancer patients frequently experience cachexia, characterized by progressive muscle wasting. BCAA supplementation has traditionally been considered potentially beneficial for improving nutritional status. Nishitani et al.’s 2013 study demonstrated that BCAAs might suppress hepatocellular cancer stem cells through mTOR activation (101), suggesting potential anti-tumor effects of BCAAs under specific circumstances. BCAAs play an important role in maintaining muscle mass by promoting protein synthesis and inhibiting degradation through mTORC1 activation.

However, based on evidence that BCAAs promote tumor growth, excessive supplementation may pose risks. High-BCAA diets promoted hepatocellular carcinoma development in mice (94). This contradiction places BCAA supplementation strategies in cancer patients in a dilemma: nutritional support is needed to maintain muscle mass, but excessive supplementation may “feed” tumors.

BCAA management in cancer patients requires individualized precision nutrition strategies, considering factors including: (1) tumor type and stage; (2) nutritional status and degree of muscle wasting; (3) treatment phase; (4) metabolic phenotype and BCAA levels; (5) tumor molecular characteristics (e.g., BCAA catabolic enzyme expression). Future research should integrate these factors to develop individualized nutrition-metabolic intervention strategies (60, 101).

### 4.6 Other potential risks

#### 4.6.1 Renal health risks

High-protein diets may increase renal metabolic burden. In patients with renal insufficiency, excessive protein or BCAA intake may accelerate renal function deterioration. BCAA metabolism generates ammonia, which may accumulate in hepatic dysfunction leading to hyperammonemia. Additionally, BCAA-induced insulin resistance and metabolic syndrome indirectly increase chronic kidney disease risk.

#### 4.6.2 Controversies in neurodegenerative diseases

The relationship between BCAAs and neurodegenerative diseases remains controversial (102). Mechanisms supporting harmful effects include: autophagy inhibition (32), promotion of mitochondrial dysfunction and oxidative stress, and interference with neurotransmitter synthesis (103). However, evidence also suggests BCAAs may possess neuroprotective properties by supporting neuronal energy metabolism and protein synthesis to maintain neuronal function; this area requires further research clarification (104).

#### 4.6.3 Unknown risks of long-term supplementation

Although BCAA supplementation is generally safe in the short term, the risks of long-term high-dose use have not been adequately studied. Potential risks include: (1) sustained mTORC1 overactivation may promote aging; (2) impaired metabolic flexibility; (3) alterations in gut microbiota composition. Excessive BCAAs may interfere with the transport and metabolism of other amino acids (such as aromatic amino acids), leading to amino acid profile imbalance and nutritional dysregulation.

The “dark side” of BCAAs primarily manifests as potential harm to multiple organ systems at pathological levels. From insulin resistance, chronic inflammation, cardiovascular disease, and MASLD to cancer, BCAA metabolic dysregulation participates in the pathogenesis and progression of various metabolic diseases. Core mechanisms underlying these effects include mTORC1 pathway overactivation, mitochondrial dysfunction, inflammatory activation, and metabolic intermediate accumulation.However, it must be emphasized that the harmful effects of BCAAs primarily occur in pathological states with impaired metabolic function, whereas at physiological levels in healthy individuals, BCAAs are essential nutrients. Therefore, the dual nature of BCAAs as both “angel” and “devil” fundamentally reflects differences in metabolic health status rather than inherent problems with BCAAs themselves. The key dimensions of this context-dependent duality—including mTORC1 activation state, circulating BCAA levels, metabolic context, molecular effects, and disease-specific clinical implications—are comprehensively summarized in **Table 1**. Future research and clinical applications must seek balance between maintaining basic nutritional requirements and avoiding pathological accumulation.

**Table 1:** The “angel” and “demon” faces of BCAAs: a context-dependent framework.

Dimension	“Angel” face	“Demon” face
mTORC1 state	Physiological/transient activation: supports protein synthesis and regulated autophagy (27).	Chronic/pathological hyperactivation: drives IRS-1 serine phosphorylation and autophagy suppression (14, 27).
BCAA level	Normal physiological range (fasting plasma ~200–400 μmol/L); acute postprandial rise following protein intake (10)	Chronically elevated fasting levels (>400–600 μmol/L) reflecting impaired peripheral catabolic disposal rather than excess dietary intake (10, 14)
Metabolic context	Insulin-sensitive state; adequate mitochondrial oxidative capacity; active BCKDH flux (14, 35)	Insulin-resistant state; impaired BCKDH flux (elevated BCKDK-mediated phosphorylation); mitochondrial dysfunction; chronic low-grade inflammation (14, 35, 114)
Primary molecular effects	Muscle protein synthesis ↑ via S6K1/4E-BP1 phosphorylation; nitrogen balance maintained; immune substrate provision (27, 47, 49)	IRS-1 serine phosphorylation → insulin resistance; BCKA accumulation → direct Akt inhibition; BCAC accumulation → mitochondrial ROS ↑; NF-κB/NLRP3 activation (14, 20, 32, 79)
Catabolic intermediates	BCKAs and BCACs efficiently cleared via active BCKDH; no pathological accumulation (19, 35)	BCKAs accumulate: directly inhibit insulin signaling (20); BCACs accumulate: impair fatty acid oxidation and mitochondrial membrane function (14, 35)
Key disease settings (beneficial)	Decompensated cirrhosis with low Fischer ratio/sarcopenia/protein-energy malnutrition; cancer cachexia; post-surgical recovery; elderly sarcopenia (9, 30, 53, 66, 71, 107)	—
Key disease settings (harmful/cautionary)	—	Obesity; T2DM; early/non-cirrhotic MASLD; metabolic syndrome; CVD risk elevation (10, 11, 14, 85–87, 89, 91).
Clinical intervention: supplementation	Recommended: RCT and guideline-supported in decompensated cirrhosis (Grade A) and sarcopenia; leucine-enriched oral formulations 12–14 g/day ≥6 months (9, 30, 71); Cochrane meta-analysis: HE risk ↓ (RR 0.73, 95% CI 0.61–0.88; NNT = 5) (66)	Contraindicated or not recommended: in patients with elevated fasting circulating BCAAs and documented insulin resistance (14, 36, 115)
Clinical intervention: non-supplementation	—	Lifestyle modification; exercise (↑ BCAA oxidation, ↓ circulating BCAAs) (78, 97); dietary BCAA moderation (137); targeting BCKDK/gut microbiota (preclinical) (36, 129)
Evidence level	RCT + international guidelines: cirrhosis/HE (9, 30, 66, 71); sarcopenia meta-analysis (35 RCTs) (53)	Prospective cohort studies + mechanistic/animal studies; fewer large intervention RCTs; causality partially supported by Mendelian randomisation (11, 110, 139)
Key unresolved questions	Optimal dose, duration, and formulation for sarcopenia; survival benefit in cirrhosis requires confirmation in larger trials	Whether elevated BCAAs are causal or consequential in T2DM/CVD (77); long-term safety of BCKDK inhibition in humans (21)

## 5 Determinants of the dual effects of branched-chain amino acids

### 5.1 Dose-response relationship

#### 5.1.1 Therapeutic window

The biological effects of BCAAs demonstrate significant dose-dependent characteristics, with a well-defined “therapeutic window.” Within this range, BCAAs exert beneficial effects, while exceeding or falling below this range may produce adverse outcomes (105). Within the physiological concentration range (plasma BCAA concentrations approximately 200-400 μmol/L), BCAAs primarily perform normal physiological functions, including serving as protein synthesis substrates and participating in energy metabolism and signal transduction. A study in healthy adults demonstrated that a single oral dose of 7g BCAAs significantly elevated plasma BCAA levels within 2–4 hours without inducing metabolic disturbances (106). Therefore, moderate BCAA supplementation (typically 5-20g per day) can provide therapeutic benefits in specific populations.

In patients with liver cirrhosis, daily supplementation of 7.2g BCAAs for 24 weeks significantly improved muscle mass, muscle strength, and liver function, while reducing the incidence of liver-related events (107). In elderly populations, leucine-enriched amino acid supplementation combined with exercise training improved physical function and maintained lean body mass (108).

However, when BCAA concentrations rise to pathological levels (>600 μmol/L), harmful effects may ensue. Studies have shown that high concentrations of BCAAs (20 mM) can inhibit mitochondrial biogenesis and oxidative gene expression in myotube cells, particularly under insulin-resistant conditions (109). High-BCAA diets (1.5 times the standard dose) promoted obesity-related metabolic disorders and tumorigenesis in animal models (94).

#### 5.1.2 Individual variability in dose-response curves

The dose-response relationship of BCAAs is non-linear and exhibits significant inter-individual variability, which is modulated by multiple factors. For instance, genetic polymorphisms in BCAA metabolic enzymes are important factors influencing individual BCAA responsiveness. Genome-wide association studies (GWAS) have identified multiple genetic loci associated with plasma BCAA levels, with the rs1440581 polymorphism upstream of the PPM1K gene showing the strongest association (110). Each allele of this variant can alter plasma leucine levels by 0.08 standard deviations and is associated with an 85% increased risk of type 2 diabetes (110).

BCAT2 gene variations also affect individual BCAA metabolic capacity. Mice with adipose tissue-specific Bcat2 knockout showed resistance to high-fat diet-induced obesity, exhibiting enhanced browning of white adipose tissue and increased thermogenic capacity (111). This suggests that individuals carrying different BCAT2 variants may have significantly different metabolic responses to BCAA supplementation.

Furthermore, an individual’s baseline metabolic state significantly influences the direction of BCAA effects. In insulin-sensitive individuals, BCAA supplementation typically does not induce metabolic disturbances; however, in individuals with existing insulin resistance, the same dose of BCAAs may exacerbate metabolic abnormalities (112). Studies have shown that BCAA infusion has limited effects on insulin sensitivity in normal mice but significantly impairs insulin signaling in high-fat diet mice (76).

Mitochondrial functional status is also a critical modulating factor. In individuals with normal mitochondrial function, BCAAs can be efficiently oxidized and utilized; however, under conditions of mitochondrial dysfunction, BCAA metabolic intermediates accumulate, producing toxic effects (113). Research indicates that insulin-resistant patients exhibit decreased mitochondrial oxidative capacity in skeletal muscle and downregulated BCAA catabolic enzyme expression, leading to accumulation of BCAAs and branched-chain acylcarnitines (114).

Finally, different disease types and disease stages exhibit significant differences in BCAAs requirements and tolerance. In patients with liver cirrhosis, the benefits of BCAA supplementation increase with disease severity, with Child-Pugh class C patients showing the most pronounced benefits (107). Conversely, in patients with metabolic syndrome, BCAA levels positively correlate with disease severity, and restricting BCAA intake may be more beneficial (115).

### 5.2 Critical role of metabolic status

#### 5.2.1 Insulin sensitivity

Insulin sensitivity is a core determinant of BCAA effects, with its status directly influencing BCAA metabolic flux and effect direction. Under insulin-sensitive conditions, insulin effectively stimulates skeletal muscle BCAA uptake and oxidation, promotes BCKDH complex activity, and accelerates BCAA catabolism (14). At this time, BCAAs primarily exert beneficial effects by promoting protein synthesis, inhibiting protein degradation, and supporting energy metabolism. Studies have shown that in healthy individuals, moderate BCAA supplementation can enhance exercise-induced muscle protein synthesis and improve exercise performance (49).

On the other hand, insulin also exerts long-term effects through regulation of BCAA metabolic enzyme expression. Following an oral glucose tolerance test (OGTT), skeletal muscle PPM1K expression is significantly upregulated in insulin-sensitive individuals, promoting BCKDH activation and BCAA oxidation (110). This metabolic flexibility ensures that BCAAs do not excessively accumulate.

In contrast, under insulin-resistant conditions, multiple mechanisms lead to BCAA metabolic dysfunction and harmful effects. First, insulin’s stimulatory effect on BCAA catabolism is weakened, while its inhibitory effect on protein degradation is lost, resulting in increased BCAA appearance rate (14). Second, BCAA catabolic enzyme expression is systemically downregulated in adipose tissue and liver, particularly with the BCKDH complex persistently maintained in a phosphorylated (inactivated) state (39). Simultaneously, accumulated BCAAs and their metabolic intermediates exacerbate insulin resistance through multiple pathways. Persistently elevated leucine over-activates the mTORC1/S6K1 pathway, leading to IRS-1 serine phosphorylation and impaired insulin signaling (14, 35). Accumulation of branched-chain keto acids (BCKAs) inhibits insulin-induced Akt phosphorylation, reducing glucose uptake and mitochondrial oxygen consumption (116). Furthermore, the valine metabolite 3-HIB promotes transendothelial fatty acid transport, increasing accumulation of lipotoxic intermediates in skeletal muscle and further impairing insulin signaling (23).

Recent research has revealed mechanisms by which BCAAs acutely induce insulin resistance. A single BCAA infusion can acutely elevate blood glucose and plasma insulin levels. Continuous BCAA infusion during hyperinsulinemic-euglycemic clamp significantly impairs whole-body insulin sensitivity, especially hepatic sensitivity (76). This acute effect provides a mechanistic explanation for how BCAAs disrupt glucose metabolism in the long term.

#### 5.2.2 Mitochondrial function

Mitochondrial functional status is a key determinant of the dual effects of BCAAs, directly affecting BCAA oxidative capacity and metabolic product fate. Under conditions of normal mitochondrial function, BCAAs can be efficiently oxidized, generating ATP and providing carbon skeletons for the tricarboxylic acid cycle. Physiological concentrations of BCAAs can even promote mitochondrial biogenesis and enhance oxidative capacity (13). Athletes, due to their strong mitochondrial oxidative capacity, do not experience metabolic intermediate accumulation and insulin resistance even with relatively high-dose BCAA supplementation (78).

Brown adipose tissue (BAT) plays an important role in BCAA clearance. Cold exposure significantly reduces plasma BCAA levels, which is associated with increased active uptake and oxidation of BCAAs in BAT mitochondria. BCAAs are utilized in BAT mitochondria for UCP1-mediated thermogenesis; defective BCAA uptake and catabolism lead to impaired BCAA clearance and abnormalities in lipid/glucose metabolism (114).

Mitochondrial dysfunction is a core pathological mechanism in obesity and type 2 diabetes, leading to decreased BCAA oxidative capacity and accumulation of incomplete oxidation products (117). In skeletal muscle of insulin-resistant patients, mitochondrial oxidative phosphorylation (OXPHOS) capacity is reduced, BCAA catabolic enzyme expression is downregulated, leading to accumulation of BCAA metabolic intermediates such as C3 and C5 acylcarnitines (114).

High concentrations of BCAAs, in turn, exacerbate mitochondrial dysfunction, forming a vicious cycle. Studies have shown that 20 mM BCAA treatment can inhibit expression of mitochondrial biogenesis markers (Ppargc1α, Cox5a, Atp5) and glycolytic enzymes in myotube cells (109). Myocyte-specific inhibition of OXPHOS accelerates BCAA catabolism, leading to excessive acetyl-CoA production and lipogenesis, which then inhibits mitochondrial respiration through acetylation-mediated mechanisms, increases reactive oxygen species (ROS) production, and promotes inflammatory responses (75).

Studies with the BCKDK inhibitor BT2 provide evidence that restoring BCAA metabolism can improve mitochondrial function. BT2 treatment promotes BCKDH activity, reduces extracellular BCAA levels, while enhancing mitochondrial proton leak and uncoupling, improving metabolic health (36, 118). This suggests that targeting BCAA catabolism may be an effective strategy for improving mitochondrial dysfunction.

#### 5.2.3 Inflammatory status

Chronic inflammation can inhibit the expression and activity of BCAA catabolic enzymes through multiple mechanisms. Pro-inflammatory cytokines such as TNF-α and IL-6 downregulate transcription of BCAA metabolism-related genes and impair BCAA oxidation by activating BCKDK to increase phosphorylation (inactivation) of BCKDH (14). In obesity-associated inflammatory states, BCAA catabolic enzyme expression is systemically decreased in adipose tissue (39). Inflammation-induced insulin resistance also indirectly affects BCAA metabolism. Inflammation activates stress kinases such as JNK and IKK, leading to IRS-1 serine phosphorylation and impaired insulin signaling, while insulin is an important regulator of BCAA metabolism (14).

On the other hand, accumulated BCAAs and their metabolic products can activate inflammatory pathways, exacerbating inflammatory responses. In db/db diabetic mice, BCAA supplementation led to elevated BCKA levels, promoting excessive macrophage activation. BCKA treatment increased production of pro-inflammatory cytokines (MCP-1, TNF-α) (75). For example, elevated BCAA levels promote secretion of high mobility group box 1 (HMGB1), which is an activator of M1 macrophages (75). This leads to polarization of adipose tissue macrophages toward the pro-inflammatory M1 phenotype, releasing large amounts of pro-inflammatory mediators, forming a vicious cycle of inflammation-metabolic disorder.

BCAA metabolic dysfunction-induced mitochondrial dysfunction and ROS production are also important mechanisms for inflammation exacerbation. ROS can activate NF-κB and NLRP3 inflammasomes, promoting maturation and release of pro-inflammatory cytokines such as IL-1β (79). Interestingly, NF-κB has dual roles in inflammation regulation, both initiating inflammation and negatively regulating NLRP3 inflammasome activation through induction of p62 accumulation and mitophagy (82).

### 5.3 Disease type and stage

#### 5.3.1 Acute stress vs. chronic metabolic diseases

Under acute disease, trauma, or surgical stress conditions, the body is in a hypercatabolic state, with BCAAs being extensively consumed for energy generation and acute phase protein synthesis. At this time, BCAA supplementation can improve nitrogen balance, support immune function, and promote tissue repair (119). For instance, in patients with brain injury, BCAA supplementation has demonstrated neuroprotective and reparative effects. Branched-chain amino acids serve as the major nitrogen donor for glutamate synthesis, playing a buffering role in post-traumatic brain injury glutamate elevation, with leucine contributing nearly 50% of nitrogen in the brain (106). A single oral dose of BCAAs can maintain elevated plasma levels for several hours, providing feasibility for acute treatment (106).

Conversely, in chronic metabolic diseases such as obesity and type 2 diabetes, the body is typically in a state of nutritional overload, with BCAA circulating levels already elevated. At this time, additional BCAA supplementation may exacerbate metabolic burden. For example, BCAA supplementation in high-fat diet mice, while not altering body weight, hepatic lipid content, or glucose-lipid metabolism, did induce dynamic changes in gut microbiota (120). Therefore, impaired BCAA catabolic capacity is a key pathological feature in chronic metabolic diseases. BCAA metabolic enzyme expression is downregulated in adipose tissue and liver, BCKDH activity is reduced, leading to accumulation of BCAAs and their metabolic intermediates (39). In such cases, restricting BCAA intake may be more beneficial than supplementation. A novel dietary intervention study showed that restricting BCAA intake reduced circulating BCAA levels by 50%, improving obesity and diabetes-related metabolic disorders (115).

#### 5.3.2 Nutritional status

In patients with malnutrition, cachexia, or sarcopenia, BCAA supplementation can improve nutritional status, maintain muscle mass, and improve prognosis. Patients with liver cirrhosis and sarcopenia receiving 24 weeks of BCAA supplementation (7.2g/day) significantly improved muscle mass, muscle strength, and physical performance, and improved liver function markers and prognosis (107). In elderly individuals, exercise combined with leucine-enriched BCAAs and vitamin D supplementation improved physical test scores and maintained lean body mass, while the non-supplemented group experienced lean body mass decline (108). This suggests that BCAA supplementation has important value in elderly populations at nutritional risk. This benefit is specific to advanced cirrhosis with documented malnutrition and reduced Fischer ratio. In contrast, for patients at earlier non-cirrhotic stages of MASLD/MASH with elevated fasting circulating BCAAs, supplementation is not appropriate; the stage-stratified framework governing this distinction is detailed in Section 6.3.3.

Cancer cachexia patients face special challenges. While BCAAs are beneficial for maintaining muscle mass, they may promote tumor growth. Individualized assessment of nutritional status, tumor type, and metabolic characteristics is crucial for formulating rational BCAA supplementation strategies (99).

In nutritional excess and obesity states, BCAA circulating levels are typically already elevated, reflecting BCAA metabolic dysfunction rather than insufficient intake (72). At this time, restricting BCAA intake may improve metabolic health. Epidemiological studies have shown that dietary BCAA intake in children and adolescents exhibits a dose-dependent positive correlation with high LDL cholesterolemia risk. Each 1g/day increase in isoleucine, leucine, and valine intake increased high LDL cholesterolemia risk by 30%, 11%, and 16%, respectively (121). This suggests that BCAA intake balance should also be monitored in pediatric populations.

However, BCAA restriction requires caution to avoid nutritional deficiency. A study in healthy adults showed that short-term dietary BCAA reduction could decrease postprandial insulin secretion, improve white adipose tissue metabolism, and gut microbiome composition (115). However, the safety and efficacy of long-term restriction require further study.

#### 5.3.3 Disease activity

During disease activity or acute exacerbation periods, metabolic demands increase, and moderate BCAA supplementation may be beneficial. During acute exacerbations of chronic obstructive pulmonary disease (COPD), BCAA supplementation may improve pulmonary rehabilitation outcomes (122). In acute decompensated heart failure, BCAA metabolic dysfunction worsens, and moderate supplementation may support cardiac function (91). However, supplementation during active periods requires weighing benefits and risks. During active periods of inflammatory diseases, pro-inflammatory states may inhibit BCAA metabolism, and supplementation may lead to accumulation and exacerbated inflammation (79).

During disease stable periods, excessive BCAA supplementation may produce adverse effects. In patients with metabolic syndrome, even during disease control periods, BCAA levels remain positively correlated with cardiovascular risk (85). In cancer patients during recovery periods, restricting BCAAs may suppress tumor recurrence (94).

Stable periods should focus on improving BCAA metabolism through lifestyle interventions. Exercise training can enhance BCAA oxidative capacity, reduce circulating BCAA levels, and improve insulin sensitivity without causing nutritional deficiency (78). Healthy dietary patterns such as the Mediterranean diet, while having limited effects on BCAA levels, may counteract the harmful effects of BCAAs through other mechanisms (87).

### 5.4 Genetic and epigenetic factors

#### 5.4.1 BCKDH gene polymorphisms

The BCKDH complex is the rate-limiting enzyme of BCAA catabolism, and genetic variations in this complex significantly affect individual BCAA metabolic capacity. The BCKDH complex consists of multiple subunits: E1α (encoded by BCKDHA), E1β (encoded by BCKDHB), E2 (encoded by DBT), and E3 (encoded by DLD) (123). Pathogenic variants in these genes cause maple syrup urine disease (MSUD), a rare BCAA metabolic disorder (124).

Beyond pathogenic mutations, common genetic polymorphisms also affect BCAA metabolism. Genome-wide association studies have identified multiple loci associated with plasma BCAA levels, with the PPM1K gene region showing the strongest association (110). PPM1K encodes the activating phosphatase of BCKDH, and its variants affect BCKDH activity and BCAA levels (125).

rs1440581, located 21kb upstream of the PPM1K gene, is the single nucleotide polymorphism (SNP) most strongly associated with BCAA levels. Each allele of this polymorphism alters leucine levels by 0.08 standard deviations and increases type 2 diabetes risk by 85% (110). Mendelian randomization analysis supports a causal relationship between BCAA metabolic dysfunction and type 2 diabetes (110).

Recently, a novel PPM1K pathogenic variant was discovered. An 8-year-old boy carrying a novel homozygous missense mutation c.925A>Gp.(Ile309Val) presented with mild MSUD and mildly elevated plasma BCAAs (124). This is the third reported case of PPM1K-related MSUD, further expanding the clinical and genetic spectrum of this gene (124).

Functional studies of PPM1K variants have revealed regulatory mechanisms. PPM1K-deficient fibroblasts exhibited only 14% of normal BCKDH activity, and transfection with wild-type PPM1K restored 35% of normal activity. Additionally, PPM1K-deficient cells showed a 2-fold increase in ROS levels, suggesting increased metabolic stress (125).

#### 5.4.2 Epigenetic regulation of metabolic enzymes

DNA methylation and histone modifications regulate transcription of BCAA metabolic genes. In obese and diabetic patients, increased DNA methylation in the promoter regions of BCAA metabolic genes in adipose tissue is associated with downregulated gene expression (39). These epigenetic changes may be induced by nutritional excess and inflammation.

Post-translational protein modifications are important regulatory mechanisms. Acetylation modifications promote BCAT2 degradation, inhibiting BCAA catabolism and exerting pro-oncogenic effects in pancreatic cancer (126). In heart failure, expression of BCAA metabolic enzymes is regulated by transforming growth factor-β-activated kinase 1 (TAK1) and p38 MAPK signaling pathways, with downregulation of the transcription factor KLF15 leading to systemic decreased expression of BCAA metabolic enzymes (91).

Phosphorylation regulation of BCKDH activity is a key post-translational modification. BCKDK phosphorylates the E1α subunit at Ser293 to inactivate it, while PPM1K dephosphorylates to activate BCKDH (125). In obesity states, hepatic BCKDH is excessively phosphorylated, inhibiting its activity (22).

Interestingly, BCKDK and PPM1K, in addition to regulating BCKDH, also regulate other substrates such as ATP-citrate lyase (ACL), integrating BCAA metabolism with lipid metabolism (22). Hepatic overexpression of BCKDK increases ACL phosphorylation, activating lipogenesis. BCKDK and PPM1K transcription levels are regulated by fructose feeding or ChREBP-β expression (22).

#### 5.4.3 Individual variability in metabolic capacity

BCAT2 gene variations affect the first step of BCAA catabolism. Adipose tissue-specific Bcat2 knockout mice showed resistance to high-fat diet-induced obesity, achieved through enhanced browning and thermogenesis of inguinal white adipose tissue (111). This suggests that individual differences in BCAT2 activity may influence obesity susceptibility and BCAA supplementation effects.AARS (encoding alanyl-tRNA synthetase) gene variations are also associated with BCAA levels. AARS mutations are associated with multiple diseases, including neurological disorders, metabolic disorders, and cancer (111). rs12149660, located at the AARS locus, is positively correlated with levels of all three BCAAs (111).

Studies of polycystic ovary syndrome (PCOS) have revealed the role of PPM1K in complex diseases: PPM1K knockout mice exhibit PCOS-like phenotypes, including elevated BCAA levels, increased androgen levels, and ovarian dysfunction (111). Dietary BCAA restriction can improve endocrine and ovarian dysfunction in Ppm1k-deficient mice (111). Phenome-wide association studies (PheWAS) have shown that PPM1K gene variants are associated with multiple complex traits (111).

These findings suggest that precision nutrition strategies based on individual genetic backgrounds may optimize BCAA management. Individuals carrying high-risk gene variants may require stricter BCAA intake restrictions, while those carrying protective variants may have better tolerance to BCAA supplementation.

### 5.5 Role of Gut Microbiota

#### 5.5.1 BCAA production by gut microbiota

The gut microbiota possesses complete BCAA biosynthetic pathways and can synthesize BCAAs from simple precursors. Metagenomic analysis has identified 14/17 gene families encoding leucine, valine, and isoleucine biosynthetic pathways (127). However, the correlation between bacterial BCAA synthesis gene abundance and serum BCAA levels is not significant after adjusting for age, sex, and body fat (127).

Furthermore, Prevotella copri has been identified as the microbe most positively correlated with elevated serum BCAAs and is more abundant in the gut of vegetarians and lacto-ovo-vegetarians (128). P. copri colonization can increase circulating BCAA levels in mice, exacerbating insulin resistance and glucose intolerance (129). Germ-free mice colonized with fecal microbiota from obese individuals recapitulate the obesity phenotype and elevated BCAA levels. PICRUSt analysis revealed enrichment of amino acid-related pathways in the gut microbiome, including valine, leucine, and isoleucine biosynthesis (128).

In addition to synthesis, gut microbiota also take up and degrade BCAAs. Bacterial BCAA inward transporter gene abundance is negatively correlated with circulating BCAAs and HOMA-IR (42). Higher bacterial BCAA inward transport driven by Faecalibacterium prausnitzii is associated with lower serum BCAA levels in early adolescents (127).

Metatranscriptomic analysis showed that BCAA degradation pathways were significantly enriched in vegetarians and lacto-ovo-vegetarians compared to omnivores (128). This suggests that although vegetarians have higher P. copri abundance (promoting BCAA synthesis), enrichment of BCAA degradation pathways may partially offset the increase in synthesis.

Gut microbiota BCAA metabolism is regulated by nutritional status. BCAA content increases significantly during fasting periods, returns to baseline after refeeding, and decreases significantly after 3 months (128). This dynamic change reflects the influence of nutritional status on microbiota-host BCAA metabolic interactions.

#### 5.5.2 Microbiota influence on BCAA metabolism

Gut microbiota influence circulating BCAA levels through both direct metabolic activity and indirect regulation of host enzyme expression.

##### 5.5.2.1 Direct modulation of BCAA levels

Microbial BCAA synthesis capacity is a key determinant of host BCAA status. *Prevotella copri* and *Bacteroides vulgatus* drive elevated circulating BCAAs in insulin-resistant individuals (130). Colonization of germ-free mice with fecal microbiota from obese donors reproduces elevated BCAA levels alongside the obese phenotype (129). Antibiotic-induced dysbiosis causes persistent alterations in BCAA-related metabolic pathways (131).

Conversely, bacterial BCAA uptake reduces circulating levels. Inward BCAA transporter gene abundance is negatively correlated with serum BCAAs and HOMA-IR (42). Higher *Faecalibacterium prausnitzii*-driven BCAA transport is associated with lower serum BCAA levels in early adolescents (128). Vegetarians show enriched microbial BCAA degradation pathways despite higher *P. copri* abundance, suggesting degradation partially offsets synthesis (129).

##### 5.5.2.2 Regulation of host BCAA metabolic enzymes

Gut microbiota metabolites regulate host BCAA catabolism. Short-chain fatty acids (SCFAs)—produced by fermentation of dietary fiber—affect BCAA metabolic gene expression through epigenetic mechanisms and stimulate GLP-1 release from intestinal L cells (132). In metabolically unhealthy individuals, skeletal muscle BCAA catabolic enzyme expression is decreased and mitochondrial function is reduced, corresponding to elevated serum BCAAs (36).

##### 5.5.2.3 Reciprocal effects of BCAA intake on microbiota

BCAA supplementation itself alters microbiota composition. In high-fat diet mice, supplementation induced dynamic changes in gut microbiota diversity across stages of insulin resistance, without affecting body weight, hepatic lipids, or glucose-lipid homeostasis (99). In hemodialysis patients, BCAA supplementation reduced the abundance of *Lacticaseibacillus paracasei* and *Bifidobacterium dentium* and significantly affected gut hormone levels including GLP-1, CCK, and PYY (133).

##### 5.5.2.4 Exercise and dietary patterns as modulators

Exercise alters gut microbiota fermentation patterns and influences diabetes prevention outcomes (97). In MASLD patients, exercise-reduced circulating BCAAs correlated with improved hepatic fat content (134). Dietary fiber intervention improves microbiota composition and reduces BCAA and other metabolite levels (135). Vegetarian dietary patterns shape a microbiome with enriched BCAA degradation capacity despite higher biosynthetic species abundance (129).

##### 5.5.2.5 Therapeutic implications

**Figure 7:**
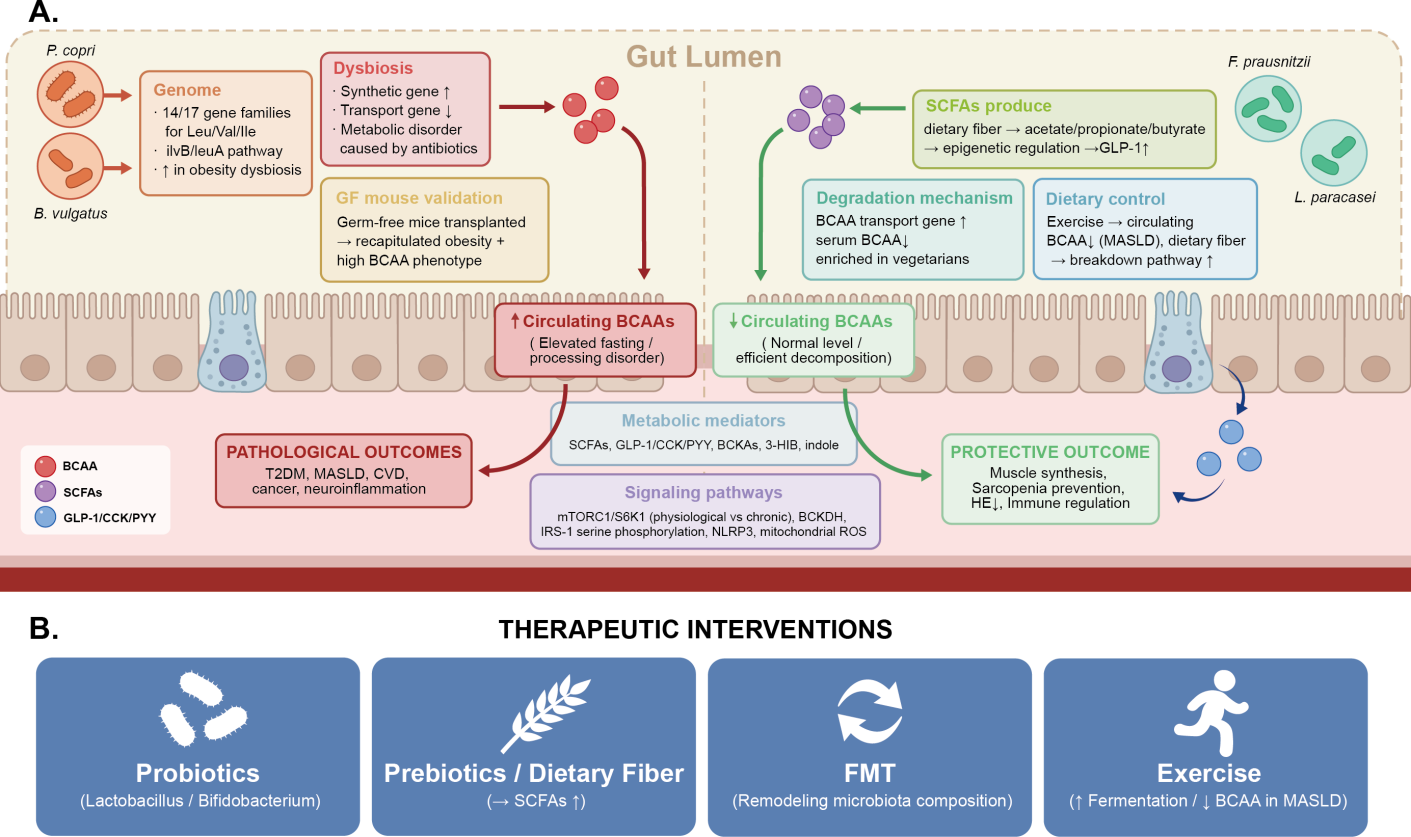
The gut microbiota–BCAA axis: mechanisms of metabolic dysfunction and health. This figure delineates the bidirectional interactions between gut microbiota and host BCAA metabolism, identifying the microbial species, metabolic pathways, and signaling mediators that determine whether the gut microbiome promotes elevation or reduction of circulating BCAA levels, and the downstream health and disease consequences. **(A)** BCAA-elevating microbiota (pathological axis). Species including *Prevotella copri* and *Bacteroides vulgatus* harbor 14/17 BCAA biosynthetic gene families (ilvB/leuA pathway) with low BCAA inward-transporter gene abundance, driving elevated circulating BCAAs. Germ-free mouse colonization with obese-donor fecal microbiota recapitulates elevated BCAA levels and metabolic disorder phenotypes. **(B)** BCAA-reducing microbiota (protective axis). Species including *Faecalibacterium prausnitzii* and *Lactobacillus/Bifidobacterium* communities possess enriched BCAA degradation pathways and inward-transporter genes, promoting circulating BCAA reduction. In vegetarians, enrichment of microbial BCAA degradation pathways partially offsets the elevated biosynthetic capacity conferred by higher *P. copri* abundance. **(C)** Metabolic mediators. The microbiome modulates host BCAA metabolism through multiple mediators. Short-chain fatty acids (SCFAs: acetate, propionate, butyrate) produced from dietary fiber fermentation regulate BCAA metabolic gene expression via epigenetic mechanisms and stimulate intestinal GLP-1 secretion. Additional mediators include GLP-1/CCK/PYY, BCKAs, 3-HIB, and indole, which activate downstream signaling pathways including mTORC1/S6K1, IRS-1 serine phosphorylation, NLRP3, and mitochondrial ROS. **(D)** Pathological versus protective outcomes. Elevated circulating BCAAs drive pathological outcomes including T2DM, MASLD, CVD, cancer, and neuroinflammation. Conversely, effective microbial BCAA degradation supports muscle protein synthesis, sarcopenia prevention, hepatic encephalopathy risk reduction, and metabolic homeostasis. **(E)** Therapeutic interventions. Four microbiota-targeted strategies are presented: ① Probiotics (*Lactobacillus/Bifidobacterium*): shift microbiome balance toward BCAA degradation; ② Prebiotics/dietary fiber (→ SCFAs↑): indirectly improve host BCAA catabolic capacity; ③ Fecal microbiota transplantation (FMT): remodel microbiota composition to alter recipient BCAA metabolism; ④ Exercise (↑ fermentation/↓ BCAA in MASLD): modulates gut fermentation patterns and enhances peripheral BCAA oxidation. Clinical implementation requires individualized microbiota profiling, as microbiome composition is shaped by diet, medications, host genetics, and environment. BCAA, Branched-Chain Amino Acid; SCFA, Short-Chain Fatty Acid; GLP-1, Glucagon-Like Peptide-1; CCK, Cholecystokinin; PYY, Peptide YY; BCKA, Branched-Chain Keto Acid; 3-HIB, 3-Hydroxyisobutyrate; mTORC1, Mechanistic Target of Rapamycin Complex 1; S6K1, Ribosomal Protein S6 Kinase Beta-1; BCKDH, Branched-Chain Alpha-Keto Acid Dehydrogenase; T2DM, Type 2 Diabetes Mellitus; MASLD, Metabolic Dysfunction-Associated Steatotic Liver Disease; CVD, Cardiovascular Disease; HE, Hepatic Encephalopathy; FMT, Fecal Microbiota Transplantation.

Targeting gut microbiota BCAA metabolism offers several intervention strategies. Probiotic or prebiotic interventions may shift the balance from BCAA synthesis toward degradation. FMT can alter recipient BCAA metabolism by remodeling microbiota composition. Dietary fiber increases SCFA production, which indirectly improves BCAA catabolism and reduces circulating levels. These approaches are illustrated in **Figure 7**.

Microbiota-based interventions face complexity: composition is shaped by diet, medications, host genetics, and environment. Individualized microbiota profiling and long-term outcome data are needed before clinical recommendations can be standardized.

## 6 Application of branched-chain amino acids in metabolic, inflammatory, and injury-related diseases

The role of branched-chain amino acids (BCAAs) in metabolic, inflammatory, and injury-related diseases demonstrates significant disease-specificity and stage-dependency. As outlined in the Introduction, this review uses “inflammatory and injury-related diseases” as a broad term encompassing metabolic disorders, immune-mediated conditions, and diseases driven primarily by chronic tissue injury, including liver cirrhosis and hepatic encephalopathy. This study systematically analyzes the dual mechanisms, clinical evidence, and precision intervention strategies of BCAAs in typical chronic diseases.

### 6.1 Metabolic syndrome

Elevated plasma BCAA concentrations serve as independent predictors of metabolic syndrome. Metabolomics analyses from the PREDIMED study identified BCAAs as part of the metabolite signature associated with metabolic syndrome incidence, with a multi-metabolite signature comprising 77 metabolites (including BCAAs) robustly associated with MetS incidence (HR: 1.56, 95% CI: 1.33-1.83) (135). The detrimental effects are mediated through the following mechanisms: chronic BCAA excess activates the mTORC1 signaling pathway, leading to increased serine phosphorylation of insulin receptor substrate-1, thereby exacerbating insulin resistance (10). Furthermore, impaired BCAA catabolism results in accumulation of 3-hydroxyisobutyrate (3-HIB), which promotes fatty acid release from adipose tissue and aggravates lipotoxicity (23).

However, for patients with sarcopenia but relatively stable metabolism, appropriate BCAA supplementation may improve muscle protein synthesis. Large-scale cross-sectional studies have shown that higher BCAA intake is associated with lower prevalence of overweight or obesity in middle-aged and older adults (136). Sarcopenic obesity presents a therapeutic dilemma for BCAA intervention: supplementation may improve muscle status but worsen metabolic dysregulation, while restriction may improve metabolism but accelerate muscle loss.

Based on this heterogeneity, BCAA intervention requires individualization: for patients with plasma BCAAs >400μmol/L and significant insulin resistance, lifestyle modifications should be prioritized to reduce endogenous levels. Studies have demonstrated that decreased BCAA consumption improves metabolic health indicators (137). For those with normal BCAAs but significant sarcopenia, low-dose supplementation (8-12g daily) combined with resistance exercise may be considered. Randomized controlled trials in elderly patients with sarcopenia have shown that leucine supplementation combined with vitamin D improves muscle mass and function (138).

### 6.2 Type 2 diabetes

Prospective cohort studies have demonstrated robust associations between baseline BCAA concentrations and future T2DM risk. The landmark Framingham Heart Study metabolomics analysis by Wang et al. showed that five branched-chain and aromatic amino acids (isoleucine, leucine, valine, tyrosine, and phenylalanine) had highly significant associations with future diabetes, with a combination of three amino acids predicting future diabetes with more than fivefold higher risk for individuals in the top quartile (11). The PREVEND prospective cohort study further confirmed that the highest BCAA quartile had a 2.8-fold increased risk of T2DM (HR 2.80, 95% CI 1.72-4.53, p<0.0001) after adjustment for multiple clinical and laboratory variables (139).

The mechanisms involve multiple levels: in peripheral tissues, chronically elevated BCAAs and their metabolites may interfere with insulin signaling; while animal experiments suggest that increasing dietary leucine intake can reduce diet-induced obesity and improve glucose and cholesterol metabolism through multiple mechanisms (140), indicating the complexity and dose-dependency of BCAA actions.

Studies on BCAA supplementation in T2DM patients show conflicting results.

Studies have demonstrated that BCAA metabolism differs significantly between health and disease states. In insulin-resistant conditions, BCAA catabolism is impaired, leading to accumulation and potential exacerbation of metabolic dysfunction; conversely, enhancing BCAA catabolism through pharmacological or dietary interventions may improve insulin sensitivity (141).Meta-analyses have shown that for elderly T2DM patients with sarcopenia, BCAA supplementation may improve body composition (142). Newly diagnosed patients with good glycemic control (HbA1c <7%) and significant sarcopenia may benefit from supplementation, while those with long disease duration and poor glycemic control require careful risk-benefit assessment.

### 6.3 Metabolic dysfunction-associated steatotic liver disease: a disease-stage-stratified framework for BCAA intervention

The role of BCAAs in liver disease is fundamentally stage-dependent; conflating early non-cirrhotic MASLD with advanced MASH-related cirrhosis leads to diametrically opposed clinical recommendations. Mechanistic underpinnings of Fischer ratio correction and ammonia metabolism are addressed in Section 3.3; this section focuses on stage-specific BCAA metabolic status and evidence-based intervention strategies.

#### 6.3.1 Early and non-cirrhotic MASLD/MASH

In early MASLD and non-cirrhotic MASH, fasting circulating BCAA concentrations are characteristically elevated, reflecting impaired peripheral catabolic capacity—reduced BCKDH activity in skeletal muscle and adipose tissue combined with insulin resistance-driven proteolysis—rather than excessive dietary intake (84, 140). Plasma valine and isoleucine concentrations are significantly elevated in MASLD independently of obesity and insulin resistance, with baseline valine levels predicting increased liver fat content during follow-up (84). The PREVEND cohort further confirmed that fasting circulating BCAAs positively correlated with fatty liver index independently of HOMA-IR, with the MASLD–T2DM association partially mediated by elevated BCAAs (mediation proportion 19.6%) (72). In this context, BCAA supplementation is not recommended: chronically elevated BCAAs sustain mTORC1/S6K1 activation, worsen hepatic insulin resistance via IRS-1 serine phosphorylation, and accelerate *de novo* lipogenesis through SREBP-1c upregulation (73, 74). Sex-dependent differences in circulating BCAA trajectories during MASLD progression should also be considered when interpreting intervention data (143).

The priority at this stage is lifestyle modification: 5–10% body weight reduction significantly improves MASLD (93, 94), and exercise training enhances skeletal muscle BCAA oxidation with BCAA reduction correlating with hepatic fat improvement (95). BCAA supplementation should be avoided in patients with elevated fasting circulating BCAAs and documented insulin resistance.

#### 6.3.2 Advanced MASH-related cirrhosis

As MASH progresses to decompensated cirrhosis, the BCAA metabolic profile undergoes a fundamental reversal: hepatic synthetic capacity declines, protein-energy malnutrition becomes prevalent, and the Fischer ratio falls as circulating BCAAs decline while aromatic amino acid levels rise due to impaired hepatic clearance (66–68, 144). This biochemical signature—low Fischer ratio, sarcopenia, and protein-energy malnutrition—defines the population in whom supplementation transitions from potentially harmful to clearly indicated.

The clinical evidence is robust. A multicenter double-blind RCT demonstrated that long-term oral BCAA supplementation significantly reduced the composite risk of death, liver transplantation, and disease progression, with event-free survival significantly improved in the BCAA group (9). BCAA granule supplementation (12 g/day for 2 years) in cirrhotic patients with hypoalbuminemia significantly improved serum albumin, reduced ascites and edema, and improved quality of life, with the greatest benefits in patients with lower baseline albumin and Fischer ratio (2). A Cochrane meta-analysis of 16 RCTs (n = 827) confirmed a significant reduction in hepatic encephalopathy risk (risk ratio 0.73, 95% CI 0.61–0.88; NNT = 5), robust in low-bias-risk trials (risk ratio 0.71, 95% CI 0.52–0.96) (4). ESPEN guidelines recommend leucine-enriched oral BCAA formulations at 12–14 g/day for a minimum of six months in decompensated cirrhotic patients with low Fischer ratio, sarcopenia, or protein-energy malnutrition, constituting a Grade A recommendation (4).

#### 6.3.3 Disease-stage decision framework

In early and non-cirrhotic MASLD/MASH, fasting circulating BCAAs are typically elevated (impaired disposal), insulin resistance is dominant, and BCAA supplementation is contraindicated—lifestyle intervention and exercise are priorities. In compensated cirrhosis with preserved synthetic function, BCAA status is variable and individualised assessment is required. In decompensated cirrhosis with malnutrition, low Fischer ratio, or sarcopenia, circulating BCAAs are characteristically reduced, protein-energy malnutrition dominates, and long-term oral BCAA supplementation carries a Grade A recommendation. Assessment of fasting plasma BCAAs, Fischer ratio, albumin, and body composition is essential before any supplementation decision in chronic liver disease.

### 6.4 Inflammatory bowel disease

Patients with inflammatory bowel disease (IBD) have high rates of malnutrition, with protein-energy malnutrition particularly common in moderate to severe cases. Systematic reviews have demonstrated that body composition alterations are prevalent in IBD patients. Bryant et al. reported that IBD patients exhibit significant changes in fat and muscle mass during both active disease and remission phases (145). Intestinal mucosal cells utilize substantial amounts of amino acids as energy sources; after mucosal barrier disruption in IBD patients, nutrient utilization efficiency decreases. Systematic reviews have demonstrated high sarcopenia prevalence in IBD patients. Nardone et al. reported that approximately 42% of adult IBD patients have myopenia, 34% have pre-sarcopenia, and 17% meet sarcopenia criteria (146). The prevalence of sarcopenia is approximately 52% in Crohn’s disease and 37% in ulcerative colitis (147). Even in clinical remission, a considerable proportion of IBD patients remain malnourished or at risk of malnutrition, with sarcopenia rates of approximately 34% (148).

Nutritional intervention for IBD requires comprehensive consideration of disease activity: Active phase: Inflammation control should be prioritized, with nutritional support primarily through enteral nutrition. Moderate amino acid supplementation (including BCAAs) should be used cautiously under professional guidance. Remission phase: Standard-dose protein and amino acid supplementation combined with resistance exercise may help improve muscle function. Body composition analysis using CT imaging at the third lumbar vertebra level is a validated method for evaluating sarcopenia severity in IBD patients (149). Perioperative period: Malnutrition is an independent risk factor for postoperative complications in IBD patients. Systematic reviews have demonstrated that sarcopenia in IBD patients is significantly associated with increased risk of requiring surgery, with three studies showing sarcopenic IBD patients had a higher probability of surgical intervention. Additionally, sarcopenia correlates with increased rates of major postoperative complications (150). For IBD patients in the perioperative period, individualized nutritional support (including adequate protein and energy) is crucial for improving outcomes.

### 6.5 Rheumatoid arthritis

The prevalence of rheumatoid cachexia ranges from 15-32% according to different diagnostic criteria, as demonstrated in a systematic review and meta-analysis by Santo et al. (151). The risk of sarcopenia is high (≥25%) in individuals with rheumatoid arthritis, and this condition is associated with increased likelihood of falls, fractures, and physical disability (152). Persistent systemic inflammation leads to elevated pro-inflammatory cytokines, directly promoting muscle protein degradation and inhibiting synthesis. The classic study by Roubenoff et al. elucidated the mechanisms of rheumatoid cachexia: cytokine-driven hypermetabolism accompanied by reduced body cell mass in chronic inflammation (153). RA patients have elevated resting energy expenditure associated with cytokine-driven hypermetabolism, but pain and activity limitations create negative nitrogen balance. Tumor necrosis factor-α is probably the central mediator of muscle wasting in rheumatoid arthritis, and is known to act synergistically with interleukin-1β to promote cachexia (154). RA patients frequently exhibit metabolic abnormalities including insulin resistance and increased prevalence of metabolic syndrome, with elevated cardiovascular disease risk (155). This presents challenges for BCAA intervention: supplementation may improve muscle status but requires vigilance regarding potential metabolic abnormalities.

Nutritional management for RA patients should be integrated into comprehensive treatment: Dietary patterns: Anti-inflammatory diets such as the Mediterranean diet may help improve overall metabolic status and disease activity. Exercise intervention: Meta-analyses of randomized controlled trials have confirmed that exercise therapy, including resistance training, significantly improves muscle mass, strength, and physical function in RA patients with sarcopenia (156). Nutritional supplementation: BCAA supplementation requires individualized assessment, with combination with exercise intervention recommended. Studies in elderly populations have shown that whey protein, amino acids, and vitamin D supplementation combined with physical activity increases fat-free mass and strength (157). Medication considerations: Glucocorticoid users experience increased protein catabolism and may require higher protein intake.

### 6.6 Malignant tumors

Cancer cachexia occurs in 50-80% of advanced cancer patients, with 20% of deaths directly attributable to cachexia. International consensus defines cancer cachexia as a multifactorial syndrome characterized by persistent skeletal muscle loss (158). Branched-chain amino acids, particularly leucine, can directly stimulate muscle protein synthesis, counteracting tumor-induced protein catabolism. Pre-clinical reviews indicate that leucine can serve as a potential therapeutic agent for muscle wasting by activating the mTOR signaling pathway to promote protein synthesis and decrease protein degradation. Multiple pre-clinical studies have demonstrated that leucine supplementation attenuates muscle wasting in tumor-bearing cachectic mice (159).

However, during tumor progression, cancer cells enhance BCAA utilization by upregulating BCAA transporters and metabolic enzymes. In human hepatocellular carcinomas and animal models of liver cancer, Ericksen et al. demonstrated that suppression of BCAA catabolic enzyme expression led to BCAA accumulation in tumors. The degree of enzyme suppression strongly correlated with tumor aggressiveness and was an independent predictor of clinical outcome. Moreover, reducing or removing BCAAs from culture media significantly suppressed mTORC1 activity and proliferation rates of HCC cell lines, and modulating BCAA accumulation regulated tumor burden and overall survival *in vivo* (94). Importantly, the effects of BCAA modulation vary across different cancer types, with some tumors showing enhanced BCAA catabolism (e.g., breast cancer) while others show suppressed catabolism (e.g., hepatocellular carcinoma) (12).

Despite theoretical concerns about tumor promotion, most clinical studies have not found that BCAA supplementation accelerates tumor progression or shortens survival. A 2023 systematic review and meta-analysis by Sideris et al. concluded that BCAA supplementation in patients with liver cancer during locoregional treatment led to higher albumin concentrations, though there were insufficient data to draw definitive conclusions on improvement in lean mass (160).

Studies in specific tumor types have provided beneficial evidence. Hepatocellular carcinoma: Long-term follow-up studies have demonstrated significant survival benefits. Nishikawa et al. showed that HCC patients receiving BCAA supplementation after radiofrequency ablation had significantly improved 1-year and 3-year overall survival rates of 94.0% and 70.0%, respectively, compared to 94.0% and 49.8% in the control group (P = 0.001). Recurrence-free survival was also significantly improved (161). A randomized trial by Nojiri et al. demonstrated that BCAA supplementation after RFA was associated with significantly lower intrahepatic recurrence rates (P = 0.036), fewer complications (P = 0.03), and significantly higher event-free survival (P = 0.04) (162). Meta-analyses have shown that BCAA supplementation improves lean body mass, grip strength, and reduces fatigue scores in cancer patients, thereby enhancing tolerance to anticancer therapies (163).

BCAA application in cancer cachexia management requires individualized strategies: during active anticancer treatment, the benefits of nutritional support generally outweigh potential risks; however, during rapid tumor progression, careful consideration is needed. Cyclical strategies, such as supplementation during chemotherapy intervals and restriction during treatment periods, may balance nutritional support with tumor promotion concerns.

The role of BCAAs across metabolic, inflammatory, and injury-related diseases demonstrates significant disease-specificity, stage-dependency, and individual variability:1. Metabolic diseases (metabolic syndrome, T2DM): Often accompanied by BCAA metabolic dysfunction, with elevated plasma BCAAs serving as predictors of disease occurrence; supplementation requires cautious consideration of metabolic risks. Liver diseases (MASLD): Require fundamentally stage-stratified intervention strategies. In non-cirrhotic MASLD/MASH, elevated fasting circulating BCAAs reflect impaired disposal, and supplementation is contraindicated. In decompensated MASH-related cirrhosis, circulating BCAAs decline and long-term oral supplementation carries Grade A recommendation evidence. Sex-dependent responses to BCAA changes should also be considered in clinical decision-making. Sex-dependent responses to BCAA changes should also be considered in clinical decision-making.2. Inflammatory diseases (IBD, RA): High rates of malnutrition and sarcopenia; appropriate nutritional support combined with exercise intervention can improve functional status. Sarcopenia prevalence is approximately 42% in IBD and 15-32% (rheumatoid cachexia) in RA.3. Cancer patients: Nutritional support is important for improving quality of life and treatment tolerance; although theoretical tumor promotion risks exist, clinical studies have not found significant harm, and specific tumor types (such as hepatocellular carcinoma) may benefit. The effects of BCAA modulation are cancer-type specific, requiring individualized approaches.

Future research directions should focus on: precision identification of biomarkers for benefiting populations, optimization of supplementation dosage and timing, evaluation of long-term safety, and development of disease-specific BCAA intervention protocols.

## 7 Conclusion

Accumulating evidence demonstrates that branched-chain amino acids (BCAAs) exert dual roles in human health and disease. This duality does not reflect experimental flaws but rather represents the inherent complexity of biological systems. From an evolutionary perspective, BCAA metabolic regulatory systems are optimized to adapt to intermittent nutrient availability: efficient utilization maintains physiological functions during nutrient scarcity, whereas accumulation under nutrient excess may induce metabolic stress (164). The modern lifestyle characterized by nutritional overabundance and sedentary behavior challenges metabolic systems originally adapted to nutrient scarcity, thereby accentuating the deleterious effects of BCAAs. The molecular basis for the dual actions of BCAAs resides in a network of context-dependent signaling processes, of which mTORC1 represents one central but not singular node. Moderate mTORC1 activation is essential for maintaining muscle mass, tissue repair, and immune function; however, in pathological states, sustained mTORC1 hyperactivation is associated with insulin resistance, cellular senescence, and autophagy suppression (165), acting in concert with BCKA accumulation, branched-chain acylcarnitine-mediated mitochondrial stress, and ROS-driven inflammatory pathway activation. The dose-response and temporal characteristics of this broader network—not mTORC1 alone—determine the duality of BCAA effects. Different tissues (skeletal muscle, liver, adipose tissue, brain) exhibit distinct characteristics in BCAA metabolism and signal transduction, with systemic effects representing the integrated outcomes of multi-organ interactions (5).

The directional effects of BCAAs depend primarily on the metabolic state of the organism rather than their absolute concentrations. In metabolically healthy states, appropriate BCAA intake supports muscle protein synthesis, maintains nitrogen balance, and promotes wound healing by moderately activating mTORC1 to sustain the dynamic equilibrium between anabolic and catabolic metabolism (47). However, under conditions of metabolic dysregulation (obesity, insulin resistance, chronic inflammation), identical BCAA levels may produce markedly different effects. Metabolic disorders lead to decreased branched-chain α-ketoacid dehydrogenase (BCKDH) activity, mitochondrial dysfunction, and elevated inflammatory cytokines, causing accumulation of BCAAs and their metabolites that trigger deleterious metabolic cascades, thereby transforming BCAAs from nutrients into metabolic stressors (166).

Based on the dual actions and metabolic state dependency, clinical applications must adhere to precision medicine principles. Precision application requires comprehensive assessment: measurement of plasma BCAA concentrations, evaluation of muscle mass and function, analysis of metabolic health indicators (insulin sensitivity, inflammatory markers, hepatorenal function), and understanding of dietary intake patterns. Metabolomic technologies can provide more comprehensive BCAA metabolic profiles, identifying metabolic bottlenecks and aberrant pathways (167). Stratified management constitutes the core of precision application. A disease-stage- and metabolic-phenotype-stratified framework for individualized BCAA intervention, incorporating key decision criteria such as fasting plasma BCAA concentration, Fischer ratio, insulin sensitivity, and body composition, is provided in **Table 2**. For patients with malnutrition, sarcopenia, or hypercatabolic states (cancer cachexia, advanced heart failure, dialysis patients), BCAA supplementation represents a clearly beneficial nutritional intervention, with typical dosages of 10–20 g per day (168). For patients with metabolic syndrome, type 2 diabetes, or metabolic dysfunction-associated steatotic liver disease, especially those with elevated plasma BCAAs, additional supplementation should be avoided. Instead, optimization of endogenous metabolism through increased physical activity, improved mitochondrial function, correction of B-vitamin deficiencies, and adjustment of dietary protein sources is recommended (169).

**Table 2:** Disease-stage- and metabolic-phenotype-stratified framework for individualized BCAA intervention.

Disease/condition	Metabolic phenotype	Fasting plasma BCAAs	Recommended strategy	Specific approach	Evidence level	Key references
Decompensated Cirrhosis (MASH-related)	Protein-energy malnutrition; low Fischer ratio; sarcopenia	Low/depleted	**Supplementation recommended**	Leucine-enriched oral BCAA 12–14 g/day ≥6 months	Grade A (Cochrane RCT, ESPEN guideline)	(9, 30, 66–68, 71, 107)
Early/Non-cirrhotic MASLD	Insulin resistance; impaired catabolic disposal	Elevated (>400 μmol/L)	**Supplementation contraindicated**	Lifestyle modification; exercise to enhance BCAA oxidation; avoid BCAA supplementation	Prospective cohort + mechanistic studies	(10, 14, 36, 74, 75, 86, 93–95, 97)
Compensated Cirrhosis	Variable synthetic function	Variable	**Individualised assessment**	Assess Fischer ratio, albumin, body composition before decision	Expert consensus	(66, 71, 107, 144)
Sarcopenia (elderly)	Reduced anabolic response; insulin-sensitive	Normal	**Supplementation beneficial**	BCAA 0.2–0.4 g/kg/day + resistance exercise; leucine-enriched preferred	Meta-analysis (35 RCTs)	(28, 46, 47, 49, 51–54, 58, 59, 108, 138)
Type 2 Diabetes with sarcopenia (HbA1c <7%, newly diagnosed)	Mild-moderate insulin resistance; muscle wasting	Mildly elevated	**Conditional supplementation**	Low-dose (8–12 g/day) + exercise; monitor insulin sensitivity	Limited RCT evidence	(11, 73, 74, 139, 141, 142)
Type 2 Diabetes (poor glycaemic control, HbA1c ≥7%)	Significant insulin resistance	Elevated	**Supplementation not recommended**	Optimise glycaemic control; exercise; dietary protein source adjustment	Prospective cohort evidence	(10, 11, 14, 35, 36, 73–76, 139)
Metabolic Syndrome without sarcopenia	Insulin resistance; elevated BCAAs	Elevated (>400 μmol/L)	**Restrict/avoid supplementation**	Lifestyle modification; weight reduction 5–10%; increase physical activity	Cross-sectional + intervention studies	(10, 14, 22, 23, 85, 87–89, 114, 115, 137)
Metabolic Syndrome with sarcopenia	Dual pathology (“sarcopenic obesity”)	Variable	**Individualised (dilemma)**	Balance muscle preservation vs. metabolic risk; exercise-first approach; multidisciplinary assessment	Expert consensus	(14, 53, 85, 115, 136)
Cancer Cachexia (non-HCC)	Hypercatabolic; muscle wasting	Low/normal	**Supplementation generally supported**	10–20 g/day; integrate with anticancer treatment; individualise by tumour type	Systematic review; tumour-type-specific data	(43, 55–57, 60, 94, 99, 159, 163)
Hepatocellular Carcinoma (post-RFA)	Post-procedural nutritional deficit	Variable	**Supplementation beneficial**	BCAA granules post-RFA; improves event-free survival and reduces recurrence	RCT evidence	(94, 101, 160–162)
IBD (active phase)	Inflammation-driven catabolism; malnutrition	Low/variable	**Cautious supplementation**	Enteral nutrition priority; moderate BCAA under professional guidance	Expert consensus	(145–150)
IBD (remission)	Sarcopenia prevalent (~34%)	Low/normal	**Supplementation with exercise**	Standard-dose protein + BCAA + resistance training	Observational + limited RCT	(53, 145–148)
Rheumatoid Arthritis	Chronic inflammation; rheumatoid cachexia	Variable	**Supplementation with caution**	Mediterranean diet + exercise + individualised BCAA; monitor metabolic status	Expert consensus + RCT for exercise	(151–157)
Athletes/Healthy adults	Insulin-sensitive; high mitochondrial capacity	Normal	**Supplementation safe; limited benefit**	Moderate supplementation (5–20 g/day) peri-exercise; complete EAA profile preferred	RCT evidence	(27, 28, 46, 47, 49, 78, 105, 106)

Key decision criteria before any BCAA intervention: Prior to initiating BCAA supplementation, the following assessments are recommended: (1) fasting plasma BCAA concentration; (2) Fischer ratio (for liver disease); (3) serum albumin and body composition (muscle mass/strength); (4) insulin sensitivity indices (HOMA-IR, HbA1c); (5) inflammatory markers (CRP, IL-6); (6) genetic risk profile where available (PPM1K rs1440581).

Bold text in the "Recommended Strategy" and "Evidence Level" columns denotes the primary clinical recommendation for each disease-condition stratum. Specifically: Supplementation recommended indicates that BCAA supplementation is supported by RCT evidence and/or guideline endorsement; Supplementation contraindicated indicates that supplementation is not recommended and may be harmful given the prevailing metabolic phenotype; Individualised assessment indicates that the clinical decision requires patient-level evaluation of Fischer ratio, albumin, body composition, and insulin sensitivity before a supplementation decision can be made; Conditional supplementation indicates that supplementation may be considered under close metabolic monitoring in a defined subpopulation; Grade A denotes the highest level of evidence based on consistent randomized controlled trial data as classified by ESPEN guidelines.

Looking forward, with the development of emerging technologies such as single-cell metabolomics and spatial metabolomics, along with deepened understanding of BCAA sensing systems, metabolite target sites, and epigenetic regulatory networks, we anticipate achieving a higher-level comprehension of the intrinsic connections among nutrition, metabolism, and health, ultimately realizing the goal of optimizing human health through precision nutritional interventions. A critical prerequisite for translating preclinical findings—particularly those involving pharmacological BCKDK inhibition—into clinical practice is the generation of long-term human safety data. Adequately powered trials are needed to establish whether sustained enhancement of BCAA catabolism can be achieved without compromising BCAA availability for essential physiological functions including muscle protein synthesis and neurological homeostasis, and to define safe dosing windows and identify patient subgroups in whom such interventions may be contraindicated. As a paradigmatic case in precision nutrition, the accumulated experience and pioneering paradigms from BCAA research will inspire and guide the development of the entire field of nutritional science.
